# Self-assembly of X-shaped antibody to combine the activity of IgG and IgA for enhanced tumor killing

**DOI:** 10.7150/thno.74903

**Published:** 2022-11-14

**Authors:** Yuexia Liang, Xin Li, Fengping Peng, Xiaohan Ye, Wei Wang, Tianyi Cen, Fan Li, Yue Lu, Zhaoyun Liu, Hui Liu, Kai Ding, Kai Ye, Yang Yu, Tianyu Ma, Sihe Zhang, Yi Huang, Yuan Wang, Xue Yang, Rong Fu, Hongkai Zhang

**Affiliations:** 1 State Key Laboratory of Medicinal Chemical Biology and College of Life Sciences, Nankai University, Tianjin 300350, PR China.; 2 Frontiers Science Center for Cell Responses, Nankai University, Tianjin 300350, PR China.; 3 Shanghai Institute for Advanced Immunochemical Studies, ShanghaiTech University, Shanghai 201210, PR China.; 4 Department of Hematology, Tianjin Medical University General Hospital, Tianjin 300052, PR China.; 5 Department of Cell Biology, School of Medicine, Nankai University, Tianjin 300350, PR China.; 6 Shanghai Tanshi Biotechnology Company, Shanghai, 201206, PR China.

**Keywords:** IgA, IgG, X-body, self-assembly, tumor-infiltrating neutrophil

## Abstract

**Rationale:** IgA can induce activation of neutrophils which are the most abundant cell type in blood, but the development of IgA as therapeutic has been confounded by its short half-life and a weak ability to recruit NK cells as effector cells. Therefore, we generated an X-shaped antibody (X-body) based on the principle of molecular self-assembly that combines the activities of both IgG and IgA, which can effectively recruit and activate NK cells, macrophages, and neutrophils to kill tumor cells.

**Methods:** X-body was generated by using a self-assembly strategy. The affinity of the X-body with the antigen and Fc receptors was tested by surface plasmon resonance. The shape of X-body was examined using negative staining transmission electron microscopy. The tumor cell killing activity of X-body was assessed *in vitro* and in multiple syngeneic mouse models. To explore the mechanism of X-body, tumor-infiltrating immune cells were analyzed by single-cell RNA-seq and flow cytometry. The dependence of neutrophil, macrophage, and NK cells for the X-body efficacy was confirmed by *in vivo* depletion of immune cell subsets.

**Results:** The X-body versions of rituximab and trastuzumab combined the full spectrum activity of IgG and IgA and recruited NK cells, macrophages, and neutrophils as effector cells for eradication of tumor cells. Treatment with anti-hCD20 and anti-hHER2 X-bodies leads to a greater reduction in tumor burden in tumor-bearing mice compared with the IgA or IgG counterpart, and no obvious adverse effect is observed upon X-body treatment. Moreover, the X-body has a serum half-life and drug stability comparable to IgG.

**Conclusions:** The X-body, as a myeloid-cell-centered therapeutic strategy, holds promise for the development of more effective cancer-targeting therapies than the current state of the art.

## Introduction

Currently, all tumor-targeting antibody therapeutics are IgG isotypes. However, resistance to IgG-based antibody treatment occurs because of impaired NK cell and macrophage activity. Therefore, there is a need to expand the anticancer arsenal for patients who do not benefit from current IgG-based therapy by exploring diverse mechanisms of action to kill tumor cells.

Other antibody isotypes, such as IgA and IgM have also been developed as cancer therapeutics 1, 2. IgA can activate neutrophils and macrophages through the engagement of FcαRI (CD89) on neutrophils and macrophages. The activation of neutrophils by IgA results in more effective tumor cell killing than the activation of neutrophils by IgG antibodies 3-7. The GD2 targeting IgA antibody improved neutrophil-mediated lysis of neuroblastoma and circumvented CDC-associated pain in a preclinical mouse model 8.

Despite the promise of IgA as a new anticancer modality, the development of IgA as a cancer therapeutic agent is confounded by IgA's short half-life and weak activation capability of NK cells 9. The combination of IgG and IgA antibodies exhibited greater efficacy in inducing tumor killing than either IgA or IgG alone. Because of the different serum half-lives of IgG and IgA, it is appealing to design one molecule to combine the activities of both IgG and IgA.

IgGA chimeric Fc displayed high affinity for FcαRI and retained binding to the activating Fcγ receptors CD64 and CD32a, leading to potent activation of both neutrophils and macrophages for cancer cell killing 5. However, IgGA did not bind to CD16a or FcRn. Heemskerk et al. generated a bispecific antibody referred to as TrisomAb 9, in which one arm targets CD89 and the other targets a tumor-associated antigen. TrisomAb effectively recruited NK cells, macrophages, and neutrophils as effector cells for the eradication of tumor cells *in vitro* and *in vivo*. Nevertheless, TrisomAb has an antigen-binding valency of 1, whereas regular antibodies have an antigen-binding valency of 2.

We developed an X-shaped antibody, referred to as the X-body, which combines the full spectrum activity of IgG and IgA. The X-body versions of rituximab and trastuzumab are more effective in recruiting neutrophils, macrophages, and NK cells for tumor killing than their IgG counterparts in both *in vitro* and *in vivo* experiments. Notably, the X-body has IgG1-like expression levels and good thermal stability, making it a promising molecule for therapeutic applications. The unconventional antibody format explores the new possibilities of multiply-specific antibodies and hold promise for the development of more effective cancer-targeting therapies than the current state of the art.

## Materials and Methods

### Mice

Human CD89 transgenic mice (cat # NM-KI-200063) and human FcRn transgenic mice (cat # NM-HU-00109) were purchased from the Shanghai Model Organisms Center, Inc. Human CD89 transgenic mice express CD89 under the CD11b promoter. Thus, the expression of CD89 is limited to mouse neutrophils and macrophages. The mice were bred and maintained in a specific-pathogen-free environment at the Department of Laboratory of Animal Science, Nankai University. All the mice had free access to regular chow and water. Age-matched 8-12 weeks old mice were used for all experiments. All animal studies were performed in compliance with the Institute Research Ethics Committee of Nankai University (Protocol Registry Number: A-2018-0306).

### Cell culture

Human Burkitt's B lymphoma cell line Ramos (ATCC, cat # CRL-1596) and Mouse Bladder Carcinoma Cell line MB49 (Merck, cat # SCC148) with human Her2 gene introduced by lentivirus were cultured in complete RPMI-1640 medium [RPMI-1640 medium (Gibico™, cat # C11875500CP) supplemented with 10% (v/v) fetal calf serum (BioInd, cat # 04-001-1A), 100 U/mL penicillin-streptomycin (PS, BioInd, cat # 03-031-1B), 10 mM HEPES buffer solution, pH 7.3 (BioInd, cat # 03-025-1B), and 1% (v/v) MEM Non-Essential Amino Acids solution (BioInd, cat # 01-340-1B)]. The human breast cell line SKBR3 (ATCC, cat # HTB-30) was cultured in McCoy's 5A medium with the same supplements as in the complete RPMI-1640 medium. HEK293F cells (Thermo Fisher Scientific, cat # R79007) were cultured in FreeStyle medium (Cat # 12338-018) for protein expression. All the cells were cultured at 37 °C and 5% CO_2_.

Neutrophils were isolated from the whole blood of healthy donors using the MACSxpress® Whole Blood Neutrophil Isolation Kit (MACS, catalog # 130-104-434). The purity of neutrophils was verified using flow cytometry by CD13 and CD15 staining and cultured in RPMI1640, 20% FBS,100 U/mL PS, HEPES pH 7.3, and MEM supplemented with 10 ng/mL GM-CSF and 50 ng/mL IFN-γ for 16-24 hours prior to use. Human PBMC were isolated from the blood of healthy human donors using density gradient centrifugation (Ficoll-Paque™ Plus, GE Healthcare). NK cells were isolated from PBMCs using the EasySep ™ Human NK Cell Isolation Kit (Stemcell, cat #17955). Human monocytes were isolated from PBMC using CD14 MicroBeads (Miltenyi Biotec, cat # 130-050-201) and then exposed to 50 ng/mL M-CSF (PeproTech, cat # AF-300-25-10) in complete RPMI-1640 medium for 14 days to generate macrophages. The study protocol was approved by the Ethical Committee of Tianjin Medical University General Hospital (Approval No. IRB2020-WZ-018).

### Protein expression and purification

The IgGs used in this study were of IgG1 isotype, and the IgAs were of IgA2 Fc without the tailpiece for dimerization and were fused to 6×His-tagged at the C-termini of the heavy chain. Genes encoding each chain of X-body, square-body, IgG and IgA were synthesized and constructed into the pTT5 vector (NovoPro, cat # V001466). The plasmids of each antibody were mixed in equal proportions. Each plasmid mixture was transfected at 1µg/mL into HEK293F cells (10^6^/mL density) cultured in Freestyle medium (Gibco™, cat # 12338-018) to express the antibody for 5 days. Similarly, the extracellular domains of human Fc receptors FcγRIIa, FcγRIIb, FcγRIIIa^F158^, and FcγRIIIa^V158^ were also expressed in a secretory manner as 6×His-tagged fusion proteins at their C-termini. His-tagged proteins were purified using HisTrap HP columns (Cat #17-5247-01; GE Healthcare). IgG, X-body and square-body were purified using HiTrap Protein A HP columns (Cat # 17-0403-01; GE Healthcare) followed by gel-filtration chromatography using a Superdex® 200 Increase column (Cat #28-9909-44; GE Healthcare). PBS pH 7.4 was used as the buffer solution for all purifications.

### Negative stain transmission electron microscopy

X-bodies or square-bodies were diluted to 200 nM in 50 mM Tris-HCl (pH 8.0) and 150 mM NaCl. A 3.5 µL aliquot of diluted antibody was applied to the formvar/carbon film of a glow discharged 300 mesh copper grid (EMS, cat # FCF300-Cu-50). After removing the excess sample, the film was negatively stained with 1% uranyl acetate and dried in the air. Micrographs were collected using a Talos F200C transmission electron microscope (Thermo Fisher Scientific) equipped with a Ceta CCD camera, with a pixel size of 1.4 Å. Two hundred images were recorded using EPU software (Thermo Fisher Scientific) with a defocus range of -1.5~-2.5 μm. CTF estimation was performed using CTFFIND4.

### Surface plasmon resonance

SPR experiments were performed using a Biacore T200 system (GE Healthcare). All assays were performed using a CM5 biosensor chip immobilized with an anti-His antibody at 20 °C. His-tagged proteins, such as CD89, FcγRIIa, FcγRIIb, FcγRIIIa^F158^, FcγRIIIa^V158^, FcγRI, FcRn, and antigen CD20, were first captured by the chip at a flow rate of 10 μL/min. Equilibrium dissociation constants (KD) were measured by injecting serial dilutions of anti-CD20 X-body, anti-CD20 IgG, or anti-CD20 IgA through the flow cells for 120 s for binding and 240 s for dissociation at a flow rate of 30 µL/min. After each cycle, the chip was regenerated using glycine-HCl pH 1.5 at a flow rate of 30 μL/min for 30 s. All binding responses were fitted using a 1:1 binding kinetic model or a steady-state affinity model in Biacore T200 evaluation software to calculate KD.

### ADCC and ADCP assay

The ADCC assays were performed at a ratio of effector cells to tumor cells of 5:1 for NK cells and 50:1 for neutrophils. Tumor cells were co-cultured with NK cells in a complete RPMI-1640 medium with the addition of the therapeutic antibodies for 4 h at 37 °C and 5% CO_2_. The tumor-cell killing effect of NK cells was monitored using the CytoTox 96® Non-Radioactive Cytotoxicity Assay kit (Promega, cat # G1780) by measuring lactate dehydrogenase (LDH) activity in the supernatants. For the ADCC assay with neutrophils as effector cells, CFSE (BioLegend, cat # 423801) pre-stained tumor cells were cocultured with neutrophils, cells were then stained with 7-AAD (Invitrogen™, cat # A1310), and analyzed by flow cytometry (BD, LSRFortessa) using FlowJo software (FlowJo, LLC). The percentage of tumor cell-killing by neutrophils was quantified using the equation 10:



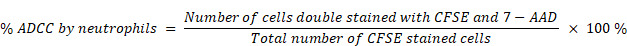



For ADCP assays, macrophages and tumor cells were labeled with CFSE and PKH26 (Sigma Aldrich, cat # mini26), respectively, and co-cultured at a ratio of 5:1 in complete RPMI-medium with antibody for 6 h at 37 °C and 5% CO_2_. And the ADCP assay is performed according to the protocol reported by de Goeij 10. Samples were analyzed by flow cytometry (BD, LSRFortessa) using FlowJo software (FlowJo, LLC). Percentage phagocytic events were quantified using the equation:



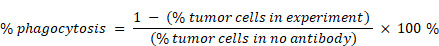



To detect the fratricide between NK cells, neutrophils and macrophages, the CFSE pre-stained NK cells were co-cultured with neutrophils or macrophages at a ratio of 1:1 in a complete RPMI-1640 medium with the addition of X-body for 4 h at 37 °C and 5% CO_2_. The sample were then stained with 7-AAD and subjected to flow cytometry. The percentage of cell death measured is expressed as the number of cells double-stained with CFSE and 7-AAD versus the total number of cells stained with CFSE. The similar experiments were performed to measure the killing of macrophages and neutrophils by the other cell types.

### Live-cell imaging

To record the induction of antibody-mediated trogocytosis by neutrophils, CFSE-labeled neutrophils and PKH26-labeled Ramos cells were co-cultured at a ratio of 8:1 in a polystyrene non-pyrogenic glass-bottom cell culture dish (Nest, Cat# 801002) containing 5 μg/mL antibody for live-cell imaging. Neutrophil-tumor cell interactions were recorded continuously using a Leica TCS SP8 laser scanning confocal microscope with hybrid photodetectors and a resonant scanner for fast image acquisition. Representative movies were generated using Fiji and Imaris software.

### Subcutaneous syngeneic tumor models

For the lymphoma model, 3×10^6^ E.g7-human CD20-Luc cells were intraperitoneally injected into FcαRI transgenic mice. Mice were intraperitoneally injected with 100 μg rituximab X-body, IgG, IgA, or PBS. Tumors were visualized by injecting 100 μL of 0.025 g/mL luciferin (Promega). Images were recorded using an IVIS Spectrum (PerkinElmer) and analyzed using Living Imaging 4.5 Software (PerkinElmer).

For the solid tumor model, 1.25×10^6^ human Her2 expressing MC38 cells or 5×10^5^ human Her2 expressing MB49 cells were injected subcutaneously into human FcαRI transgenic mice. Tumor growth was monitored using Vernier calipers and recorded. Tumor volume (mm^3^) was calculated using the equation:



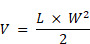



where V is the tumor volume, L represents the tumor length in mm, and W is the tumor width in mm. Tumor-bearing mice were injected intraperitoneally with antibodies in PBS (10 mg/kg) every three days for a total of five injections, as the tumor volume reached 30-50 mm^3^. Tumor volume and survival were measured every three days. Mice were sacrificed in accordance with the Animal Welfare Policy when the tumor volume reached the humane endpoint of 2000 mm^3^.

### Flow cytometry analysis of immune cells

Tumors collected from mice were dissociated with 0.5 mg/mL DNase I,1 mg/mL collagenase I, and 0.05 mg/mL dispase II in PBS. Single cells were obtained using a 40 μm cell strainer. For multicolor fluorescence-conjugated antibody staining, cells were blocked with αCD16/32 antibody (101320, BioLegend) and stained with the Zombie Aqua Fixable Viability Kit (423101, BioLegend) to distinguish dead cells. Cell surface markers were stained with specific antibodies, for 30 min at 4 °C in the dark. Intracellular markers were stained using antibodies for 45 min at 4 °C after the cells were fixed and permeabilized by using permeabilization buffer (421002, BioLegend). Cells were detected using an LSR Fortessa flow cytometer (BD Biosciences) and analyzed using FlowJo software (BD Biosciences).

Antibodies listed for cell-marker staining were purchased from BioLegend: anti-mouse CD45 PE-Cy5 (103109), anti-mouse CD3 BV510 (100234), anti-mouse NK1.1 PerCP-Cy5.5 (108727), anti-mouse Granzyme B BV421 (396414), anti-mouse CD107a PE (121611), anti-mouse CD45 PerCP-Cy5.5 (103132), anti-mouse CD11b PE-Cy5 (101210), anti-mouse F4/80 BV650 (123149), anti-mouse iNOS PE (696805), anti-mouse CD206 PE-Cy7 (141720), anti-mouse CD86 BV605 (105037), anti-mouse CD45 APC (103111), anti-mouse CD3 PerCP-Cy5.5 (100328), anti-mouse Ly6G BV605 (127639), anti-mouse CD24 FITC (101805), anti-mouse CD45 BV510 (103138), anti-mouse CD3 FITC (100204), anti-mouse NKp46 PE-Cy7 (137618), anti-mouse Granzyme B PE (372208), anti-mouse IFN-γ PE-DE594 (505846), anti-mouse CD11b pacific blue (101224), anti-mouse F4/80 PE-Cy7 (123114), and anti-mouse CD206 PE-DE594 (141732).

### Preparation for scRNA-Seq Library

Three days after the last antibody injection, tumor tissues were collected from the mice and minced. Small tumor masses were digested with 1 mg/mL Collagenase I, 0.5 mg/mL DNase, and 0.05 mg/mL Dispase II in PBS for 1 h at 37 °C with shaking. Single cells were obtained through a 70 μm cell strainer, and the cells were washed with RPMI-1640/2% FBS, followed by PBS. Excess erythrocytes were removed with the red blood cell lysis buffer (Solarbio, Beijing, PR China, cat# R1010), then the remaining cells were resuspended in RPMI-1640/2% FBS, and their viability was detected with trypan. After the removal of dead cells using the Dead Cell Removal Kit (Miltenyi Biotec, Germany, cat#130-090-101), CD45^+^ immune cells were sorted from single-cell suspensions using CD45 (TIL) Microbeads (Miltenyi Biotec, Germany, cat#130-110-618), according to the manufacturer's protocol. Cells were used for scRNA-Seq library construction when more than 85% of cells were in good condition, as determined by bench blue staining.

### Identification of Cell Types and Cluster Marker Genes in Seurat

Gene-cell matrices were imported into the R package Seurat (version 4.0.5) 11 pipeline for quality control and downstream analysis. Low-quality cells (<200 genes cells^-1^, >7,000 genes cells^-1^, >50,000 UMIs cells^-1^, >5% mitochondrial) and CD45^-^ cells were excluded. The data were first normalized using the LogNormalization function with a scale factor of 10,000. The 2000 genes with the highest variance were detected using the FindVariable Genes function. Principal component analysis (PCA) was used on highly variable genes to reduce the dimensionality of the feature space. The top 62 principal components that can explain above 90% of the variance in the data were used to perform the downstream analysis. The R package Harmony (version 1.0) 12 was used for batch correction. The main cell clusters were identified using the FindClusters function in Seurat with a resolution of 0.7. They were then visualized using t-distributed stochastic neighbor embedding (t-SNE) plots. Each main cell cluster was manually categorized into a known biological cell type, according to conventional markers. We applied DoubletFinder (2.0.3) 13 to identify artifactual libraries generated from two or more cells that entered the same microfluidic droplet and were labeled with cell barcodes. We estimated the percentage of doublets in each cluster and removed doublet clusters that contained a large number of potential doublet cells. Specifically, we removed the CD3d^+^ CSF1R^+^ cluster with a large fraction of potential T cell-macrophage doublets. The FindMarkers function in Seurat was used to identify cluster-specific marker genes or differentially expressed genes between the PBS and X-body treatment groups in an individual cluster. The average expression of the genes within each cluster was calculated, and heatmaps of gene average expression were generated using the R package ComplexHeatmap (version 2.8.0) 14.

### Pathway Enrichment Analysis

We performed gene-set enrichment analysis (GSEA) using the R package WebGestaltR 15 to identify the pathways enriched in Gene Ontology, KEGG, and Reactome. The pathway size was limited to 500 genes per pathway, and genes were ranked by log 2-fold change calculated using the Findmarkers function in Seurat. As mentioned above, 1000 permutations were used to estimate the FDR for the GSEA analysis.

### *In vivo* serum half-life determination

Human FcRn transgenic mice (n = 3) were injected intraperitoneally with a single dose of 10 mg/kg human anti-HER2 IgG1, IgA or X-body. Blood was drawn from the tail vein at the indicated time points. Human IgG1 and X-body levels were measured using ELISA. Goat anti-human IgG Fc antibody (109-006-098, Jackson) and HRP-conjugated goat anti-human IgG (H+L) antibody (109-036-088, Jackson) were used for the capture and detection of human IgG1 and X-body, respectively. Human Her2 / ErbB2 Protein (HE2-H5212, Acrobiosystems) and Anti-6× His tag® antibody [AD1.1.10 (HRP) (ab178563, Abcam)] were used for the capture and detection of human IgA, respectively.

### Statistical Analysis

Quantitative data are presented as the mean ± standard error (SEM) of at least three independent experiments. Data were analyzed using two-tailed Student's t-test. Animal survival was presented using Kaplan-Meier survival curves, and survival data were analyzed using the log-rank test. A p-value less than 0.05 (*) was considered statistically significant (*p < 0.05, **p < 0.01, ***p < 0.001).

## Results

### Design of X-body and square-body

**Figure 1:**
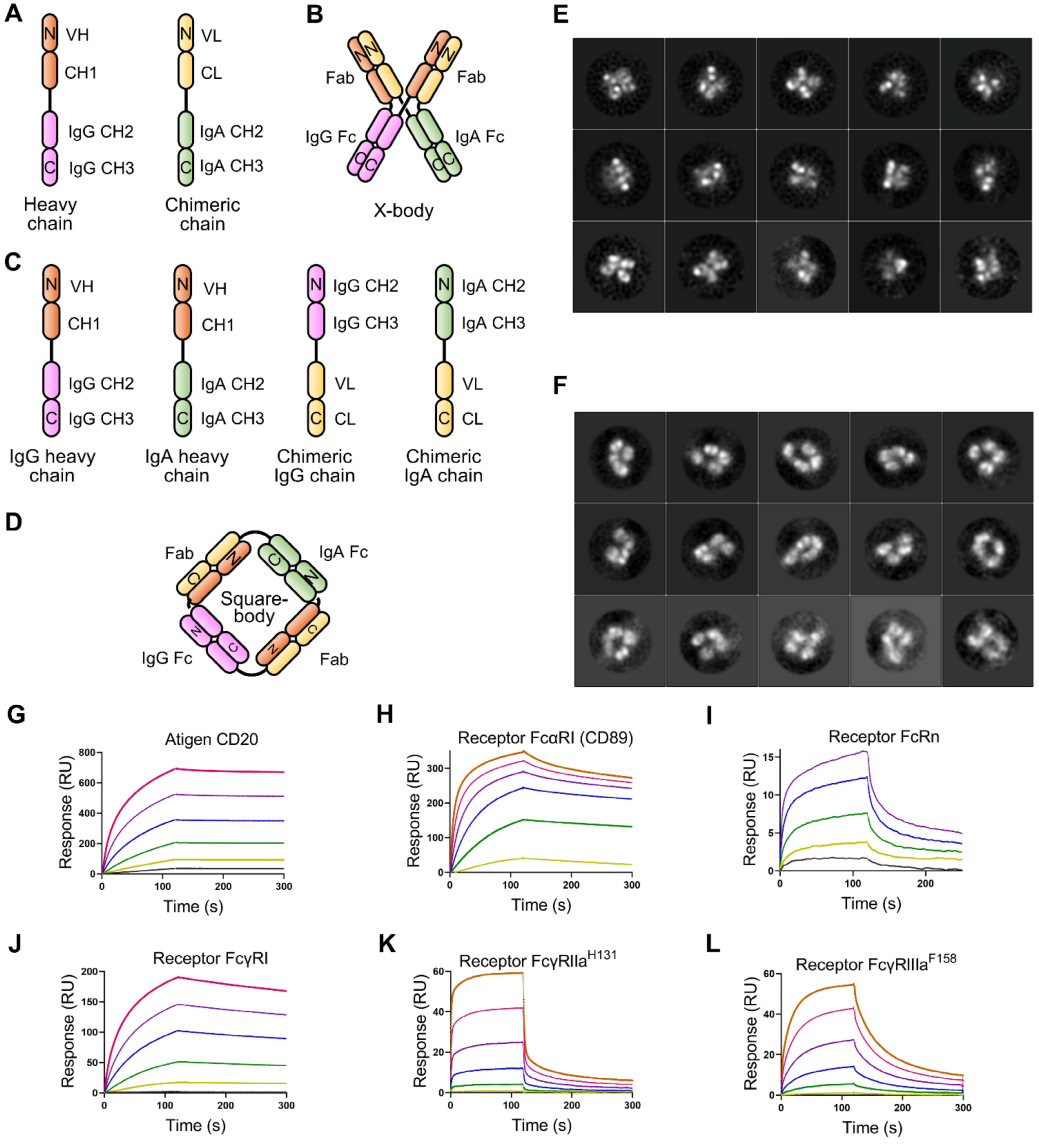
** Design and validation of X-body and square-body. A)** Schematic diagram of the heavy chain and light chain-IgA Fc chimeric chains of the X-body. **B)** Schematic of X-body. **C)** Schematic diagram of the two heavy chains and two chimeric chains to assembly the square body. **D)** Assembly of square body. The N-terminal-and C-terminal domains of each chain were labeled as N and C, respectively. Electron microscopy images of the X-body **(E)** and square body **(F)**. Through 2D class averaging, 9102 X-body particles (**E**) or 4873 square-body particles (**F**) were sorted into 15 classes. The binding of rituximab X-body to antigen CD20 **(G)** and Fc receptors, such as CD89 **(H)**, FcRn **(I)**, FcγRI **(J)**, FcγRIIa^H131^ **(K)**, and FcγRIIIa^F158^ **(L)**, as evaluated by SPR.

To effectively recruit neutrophils and macrophages and retain FcRn binding ability, we designed an X-body by fusing IgA Fc to the C-terminus of the antibody light chain and co-expressing this chimeric chain with the conventional heavy chain of IgG (Figure 1A). The assembled wild-type IgA can dimerize through its C-terminal tailpiece region, and removal of the tailpiece region can prevent dimer formation 16. Therefore, IgA2 Fc without its tailpiece region is used to construct X-body to avoid aggregation. During self-assembly of X-shaped structure consisting of two antibody Fabs, one IgA Fc, and one IgG Fc of X-body, IgG Fc and IgA Fc domains homodimerize and the VH-CH1 and VL-CL heterodimerize (Figure 1B).

We also generated a square-shaped antibody (square-body). In addition to a natural IgG heavy chain and a natural IgA heavy chain, we designed two chimeric chains. One is to fuse the antibody light chain VL-CL to the C-terminus of the IgG Fc. The other is to fuse the light chain to the carboxyl end of the IgA Fc (Figure 1C). Theoretically, these four chains could be assembled into a square-shaped molecule containing one IgG Fc, one IgA Fc, and two Fabs through homodimerization of IgG Fc and IgA Fc and heterodimerization of Fab (Figure 1D).

### Generation and validation of rituximab X-body and square-body

To validate the concept of X-body and square-body, the anti-CD20 IgG antibody rituximab was modified to X-body and square-body. To generate the X-body version of rituximab, IgA2 Fc without the tailpiece region was fused to the C-terminus of the rituximab light chain through an optimized length of G_4_S linker (Six GGGGS tandem sequence) to form the chimeric chain (Figure S1A, Table S1), which was then co-expressed with the rituximab heavy chain in 293F cells. Rituximab X-body purified by Protein A affinity chromatography and size exclusion chromatography (SEC, Figure S1A) showed a molecular weight of more than 200 kD on non-reducing SDS-PAGE and approximately 50 kD on reduced SDS-PAGE (Figure S1B), consistent with the theoretical molecular weight and the molecular weight determined by SEC (Figure S1A), indicating that rituximab X-body assembled into tetramer. The purified rituximab X-body was then subjected to negative staining transmission electron microscopy (NS-TEM) to visualize the molecule. Through 2D class averaging, 9102 of 33580 X-body particles were sorted into 15 classes (Figure 1E), representing different viewing angles of X-body molecules. The images clearly show that the rituximab X-body is X-shaped tetramer with four arms. Comparing the images of different classes, the four arms of the X-body can be in different orientations, indicating that the four arms are relatively independent and can swing freely within a certain range.

For the rituximab square-body, the rituximab light chain was fused to the C-terminus of IgG Fc or IgA2 Fc without the tailpiece region via the 6 × G_4_S linker to generate two chimeric chains. The two chimeric chains and the IgG and IgA2 versions of rituximab heavy chains were co-expressed and purified. The resulting square-body also showed a similar SEC result as the X-body (Figure S1C) and a molecular weight of more than 200 kD on non-reducing SDS-PAGE (Figure S1D). In the subsequent NS-TEM experiment, 4873 out of 64215 particles were sorted into 15 classes through 2D class averaging (Figure 1F). Unlike the X-body, a hole can be observed in the center of the molecule, providing evidence for the formation of a square-shaped molecule.

Additionally, the character and stability of the X-body were assessed. As shown in Figure S2A-C, the isoelectric point of X-body is 9.151, the purity of X-body can reach as high as 97.3%, the *T*_m_ of X-body in PBS is as high as 70 °C, and the thermal stability of X-body in other commonly used buffers is similar to that in PBS (Figure S2D). In terms of storage, the X-body was stable at 37 °C for more than 7 days (Figure S2E).

### Characterization of rituximab X-body and square-body

We examined the affinity of rituximab X-body or square-body to receptor CD89, FcγRs, FcRn, and antigen CD20 using surface plasmon resonance (SPR) with purified proteins. As shown in Figure 1G-L, the rituximab X-body not only binds to CD89 with an affinity similar to that of the IgA version of rituximab, but also retains FcRn and various FcγR binding abilities. The affinity of rituximab X-body for CD20 is equivalent to that of IgG or IgA rituximab (Table S2). The data implied that X-body simultaneously possess IgA Fc, IgG Fc, and Fabs with unimpaired function. On the other hand, the affinity of the rituximab square-body to some receptors is greatly impaired, presumably due to steric hindrance (data not shown). Therefore, in the follow-up study, we only investigated the activity of the X-body.

### CD20 targeting X-body can induce neutrophils, NK cells and macrophages mediated cytotoxicity against tumor cells

**Figure 2:**
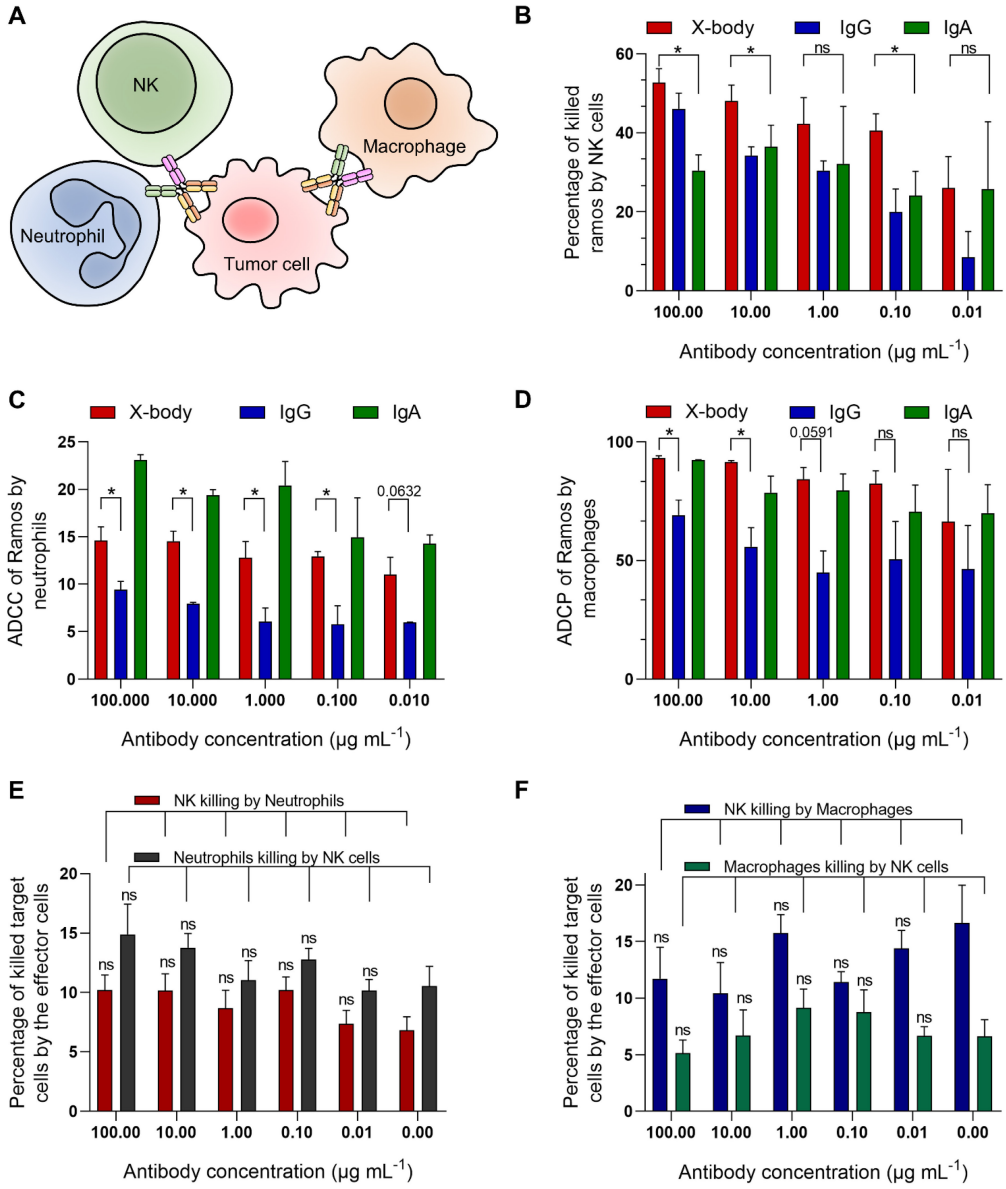
** The cytotoxicity assays of rituximab X-body. A)** Schematic illustration of the effects of X-body in cancer cell killing. **B-D)** Killing of Ramos cells by NK cells (B), neutrophils (C) and macrophages (D) in presence of rituximab X-body (red), IgG (blue) or IgA (green) at the antibody concentrations of 100, 10, 1, 0.1, and 0.01 µg mL^-1^. **E-F)** Co-culture of NK cells with neutrophils (E) or macrophages (F) in the presence or absence of rituximab X-body. "ns": statistically non-significant.

Since the X-body has both functional IgG Fc and IgA Fc, it should not only recruit NK cells and macrophages, but also recruit neutrophils as effector cells to kill tumor cells (Figure 2A). We examined ADCC or ADCP activity of rituximab X-body using Ramos tumor cells as target cells and NK cells, macrophages or polymorphonuclear cells (PMN) as effector cells. When using PMN as the effector cells, X-body treatment resulted in antibody-concentration-dependent tumor killing (Figure 2C). The X-body induced stronger tumor-killing activity at each concentration compared to rituximab IgG (Figure 2C). Furthermore, rapid engulfment of the X-body opsonized tumor cells by neutrophils was observed, whereas this process was inefficient with IgG antibody (Video S1-3). When using NK cells or macrophages as effector cells, the rituximab X-body-mediated cytotoxicity was superior to that of either rituximab IgA or IgG (Figure 2B, D). These data confirmed that X-body retained effector-cell recruitment activities of both IgG and IgA for tumor-cell killing.

Because X-body has both IgG Fc and IgA Fc, it is concerned whether X-body can pull two effector cells together and cause fratricide between themselves. To clarify this issue, we cocultured NK cells with neutrophils or macrophages in the absence or presence of the X-body. The results showed that the X-body did not cause statistically significant changes in the viability of either cell (Figure 2E, F).

### CD20 targeting X-body can clear CD20 expressing leukemia cells in a syngeneic mouse model

**Figure 3:**
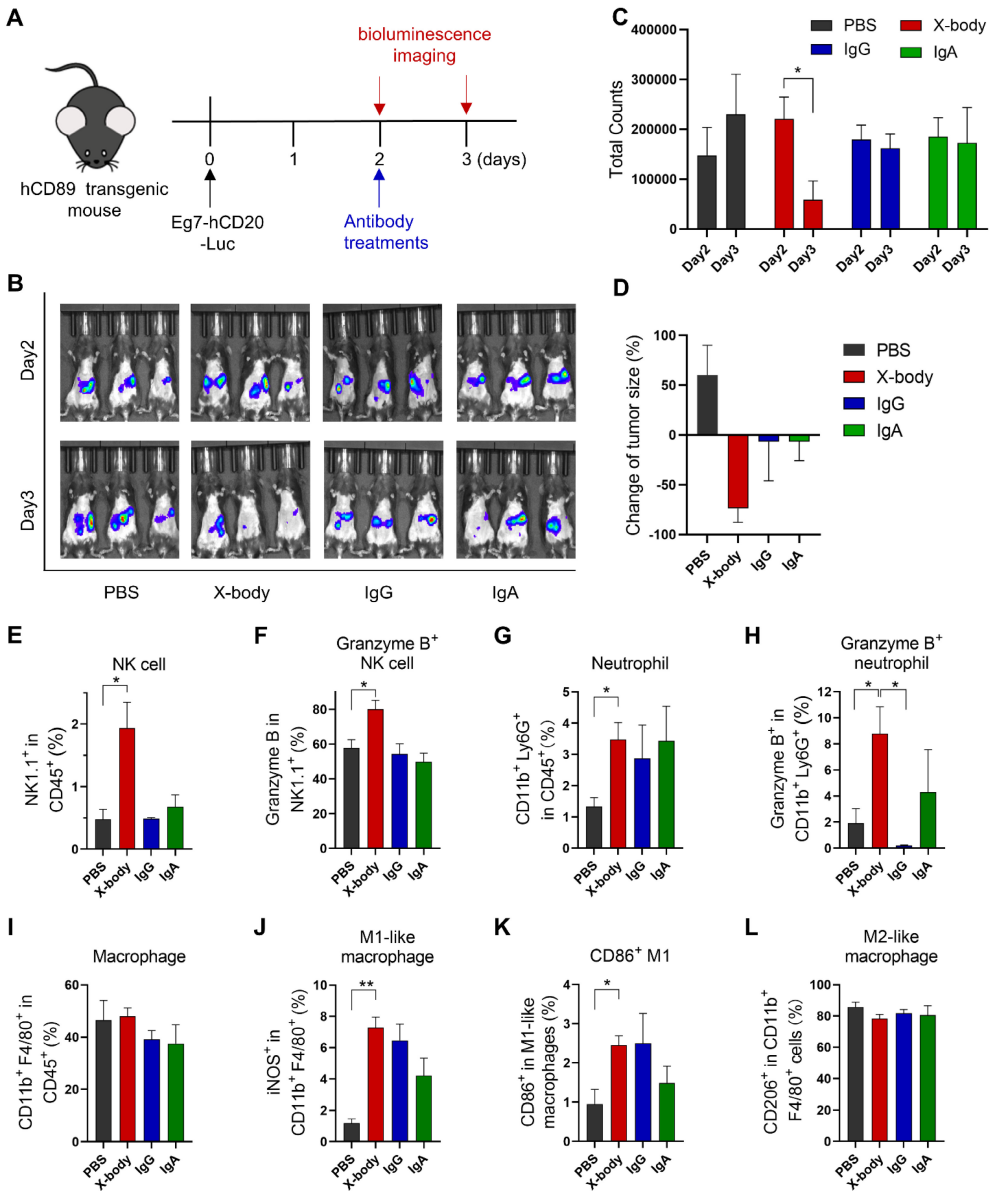
** The anticancer efficacy of rituximab X-body in the Eg7 syngeneic murine model. A)** Schematic of the treatment regimen. Eg7-hCD20-Luc tumor cells were intraperitoneally inoculated into human CD89 transgenic mice. The mice were treated with different forms of rituximab antibody. **B)** Bioluminescence imaging (BLI) of Eg7-hCD20-Luc tumor cells before treatment (Day2) and 24 hours after treatment (Day3). **C-D)** Quantification of bioluminescence signal and inhibition of cancer cells by the treatment was calculated. * p ≤ 0.05. **E-L)** Proportion and activation status of infiltrating immune cells in ascites of tumor-bearing mice. Cells were isolated from mouse ascites, stained with the indicated antibodies and analyzed by flow cytometry. The percentages of NK cells (E), neutrophils (G) and Macrophages (I) and the activation status of NK cells (F), neutrophils (H), and macrophages **(J-L)** were analyzed. Data are presented as mean ± standard error of the mean (SEM). * p ≤ 0.05.

Human CD89 transgenic C57BL/6J mice (Figure S3 for the expression of CD89 on immune cells) were intraperitoneally engrafted with mouse leukemia cells, EL4-hCD20-Luc cells, which co-express human CD20 and luciferase. Tumor-bearing mice were treated with IgG, IgA, or X-body version of rituximab one day after tumor cell inoculation (Figure 3A). The number of tumor cells was evaluated by bioluminescence analysis (Figure 3B). X-body treatment eradicated most tumor cells while the IgA or IgG treatment modestly delayed the tumor growth. The tumors progressed in mice in the PBS group (Figure 3C and D).

We analyzed the immune cells in mouse ascites after treatment using multicolor flow cytometry. As shown in Figure 3E, X-body treatment resulted in higher percentage of NK cells in ascites compared with other two treatments and more NK cells were activated upon X-body treatment in comparison with IgA treatment (Figure 3F). X-body treatment also induced more Granzyme B-expressing neutrophils than IgG treatment (Figure 3G-H) 17. Nevertheless, more M2-like macrophages were re-polarized into M1-like macrophages in all groups with the biggest phenotype shift observed in X-body treatment group (Figure 3J-L).

### HER2 targeting X-body inhibited tumor growth in MC38-HER2 tumor cell bearing syngeneic model

To verify the concept of the X-body in solid tumors, we generated an anti-HER2 trastuzumab X-body. The trastuzumab X-body showed intact antigen or IgG receptor binding affinity compared to trastuzumab IgG and acquired the CD89 binding ability of the IgA (Table 1, Figure S4). Therefore, we examined the tumor-inhibition activity of X-body in mice.

**Figure 4:**
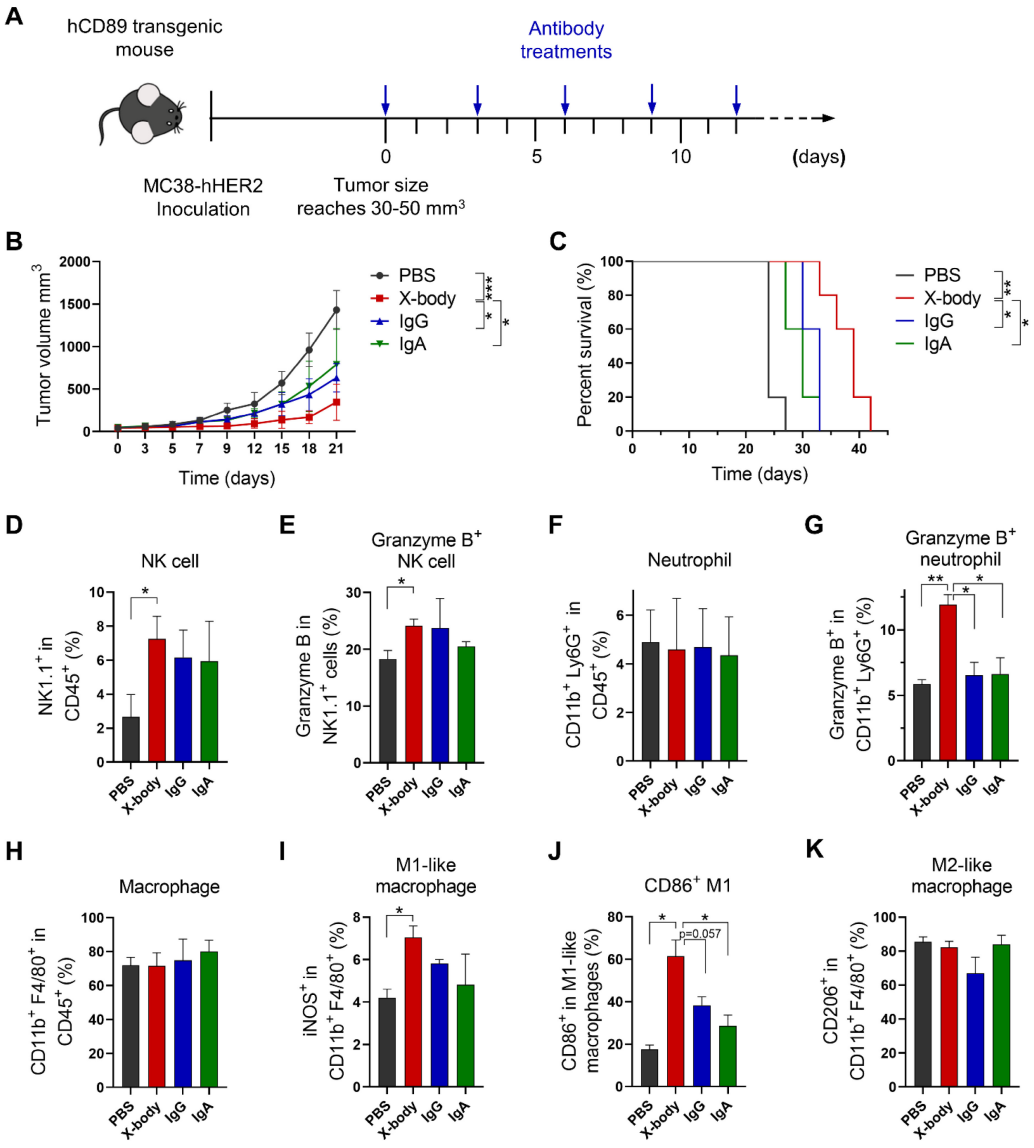
** Tumor inhibition efficacy of trastuzumab X-body in colon cancer syngeneic mouse model. A)** Schematic of the treatment regimen. MC38-HER2 cancer cells were subcutaneously implanted in human CD89 transgenic mice. When tumor reached 30-50 mm^3^, the mice were treated with different version of trastuzumab at the indicated time points. **B)** Tumor growth of mice treated with PBS, trastuzumab X-body, IgG or IgA. **C)** Kaplan-Meier survival curves of tumor-bearing mice. **D-K)** Flow cytometry analysis of the tumor-infiltrating immune cells. Tumor immune cells were isolated three days after 5 doses of drugs, stained with indicated antibodies and analyzed by flow cytometry. The percentages of NK cells (D), neutrophils (F), and macrophages (H) and the activation status of NK cells (E), neutrophils (G), and macrophages (I-K) were analyzed. Data are presented as mean ± standard error of the mean (SEM). ** p ≤ 0.01, * p ≤ 0.05.

Human HER2 expressing mouse tumor cell line MC38-HER2 was subcutaneously engrafted in human CD89 transgenic mice and the tumor bearing mice were treated with IgG, IgA, or X-body version of trastuzumab (Figure 4A). Trastuzumab X-body treatment significantly inhibited tumor growth and extended median survival (39 days for X-body treatment vs. 24 days for vehicle control) (Figure 4B, C). In contrast, treatment with trastuzumab IgG and IgA resulted in only modest tumor suppression.

In order to explore the mechanism of action of X-body version of trastuzumab, the tumor-infiltrating NK cells, macrophages and neutrophils were analyzed by using flow cytometry. X-body treatment results in higher proportion of tumor infiltrating NK cells compared with IgA treatment (Figure 4D, E). Although neither treatment changed the number of infiltrating neutrophils compared with the PBS group (Figure 4F), only treatment with X-body caused activation of neutrophils, as indicated by the expression of Granzyme B (Figure 4G). Although neither treatment had effect on the total number of tumor associated macrophages (Figure 4H), X-body treatment shifted the bias of macrophage to a more M1 -like pro-inflammatory phenotype as indicated by the expression of iNOS and CD86 (Figure 4I, J). In addition, the X-body treatment group significantly down-regulated the expression of the chemokine CCL2 (Figure S5), which is reported to promote polarization of the anti-inflammatory M2-like macrophages (Figure 4K).

### HER2 targeting X-body delayed tumor growth in Her2 expressing MB49 model

**Figure 5:**
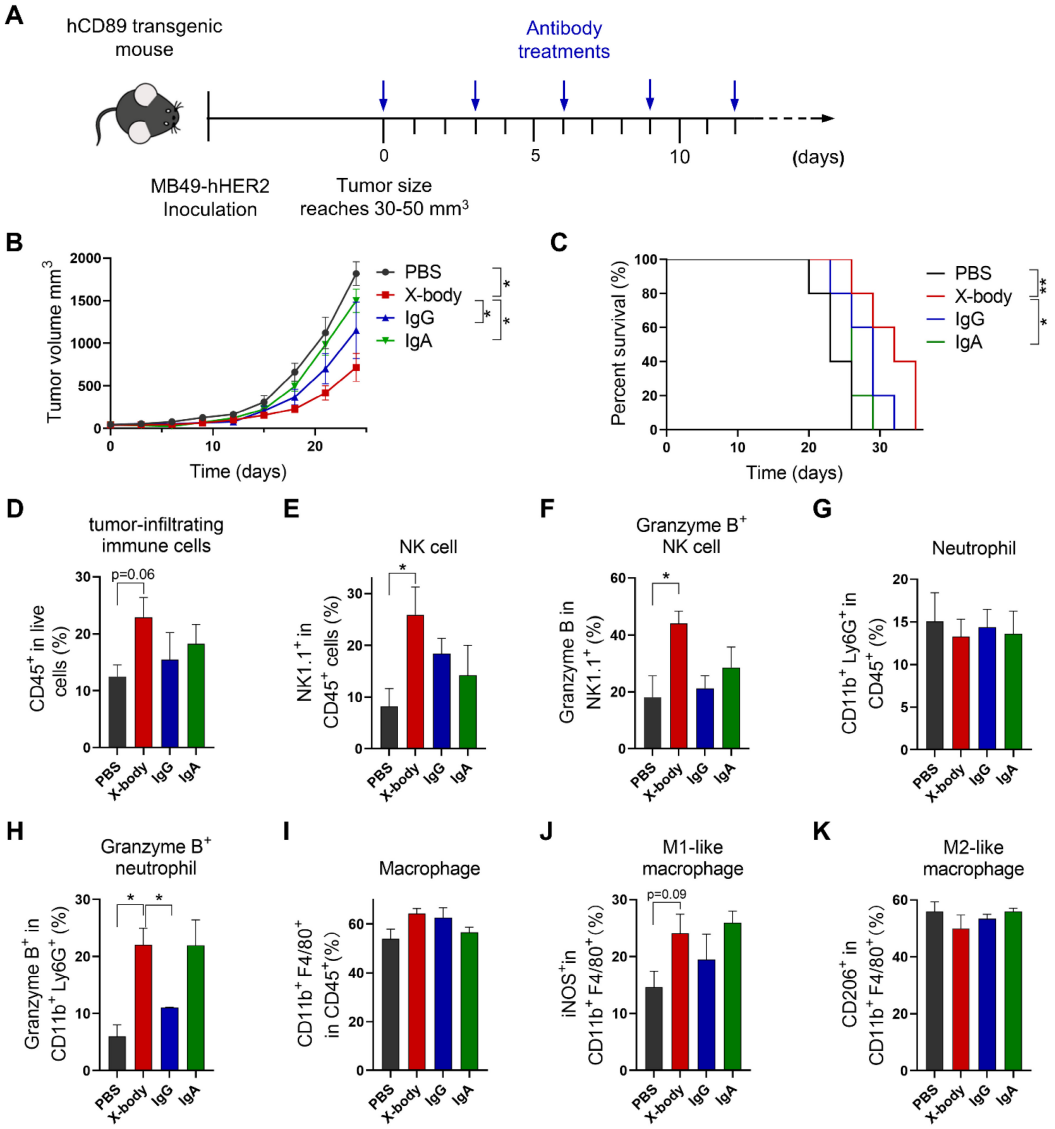
** Tumor inhibition efficacy of trastuzumab X-body in bladder cancer syngeneic mouse model. A)** Schematic of the treatment regimen. MB49-HER2 cancer cells were subcutaneously implanted in human CD89 transgenic mice. **B)** Tumor growth of mice treated with PBS, trastuzumab X-body, IgG or IgA. **C)** Kaplan-Meier survival curves of tumor-bearing mice. **D-K)** Flow cytometry analysis of the tumor-infiltrating immune cells. Tumor immune cells were isolated three days after 5 doses of drugs, stained with indicated antibodies and analyzed by flow cytometry. Proportion of tumor-infiltrating immune cells in live cells (D), the proportion of NK cells (E), neutrophils (G), and macrophages (I) in total tumor-infiltrating immune cells and the activation status of NK cells (F), neutrophils (H), and macrophages (J and K) were analyzed. Data are presented as mean ± standard error of the mean (SEM). ** p ≤ 0.01, * p ≤ 0.05.

We also evaluated efficacy of X-body in MB49 tumor cell-bearing mouse model which is considered to have a cold tumor phenotype (Figure 5A). Trastuzumab X-body produced anti-tumor activity with 9 days tumor growth delay compared to vehicle group, and X-body demonstrated superior anti-tumor activity to IgA and IgG (Figure 5B). Although the response to X-body treatment seems superior to the response to IgG treatment, we didn't observe significant difference between the median survival of X-body group and IgG group (Figure 5C). The limited activity of X-body in MB49 syngeneic mouse model provides significant room for improvement with combination therapy.

To identify the immune cell types that involved in the inhibition of tumor growth by X-body, NK cells, macrophages, or neutrophils were depleted with anti-mouse NK1.1, anti-mouse CSF1R, or anti-mouse Ly6G antibodies during X-body treatment (Figure S6A). Depletion of NK cells, macrophages, or neutrophils resulted in reduced tumor regression after X-body treatment, indicating that all three immune cells play critical roles in tumor-cell killing by trastuzumab X-body (Figure S6B, C).

### X-body increased the infiltration and activation of NK cells and neutrophils but did not reprogram macrophages to the proinflammatory phenotype

To investigate the mechanism of X-body, we analyzed tumor-infiltrating immune cells following treatment. X-body, IgA, and IgG antibodies increased the number of tumor-infiltrating immune cells (Figure 5D). The infiltration and activation of NK cells (Figure 5E, F) were induced by treatment with X-body, IgG, or IgA, with the strongest NK cell activation observed in the X-body treatment group. The proportion of neutrophils was not significantly changed by treatment with X-body, IgA, or IgG (Figure 5G). However, the proportion of neutrophils expressing Granzyme B was significantly increased (Figure 5H). The macrophages in each group were still predominately M2-like macrophages (Figure 5I-K), suggesting that induction of M2-to-M1 repolarization with other drugs may further improve the efficacy of the X-body treatment.

**Figure 6:**
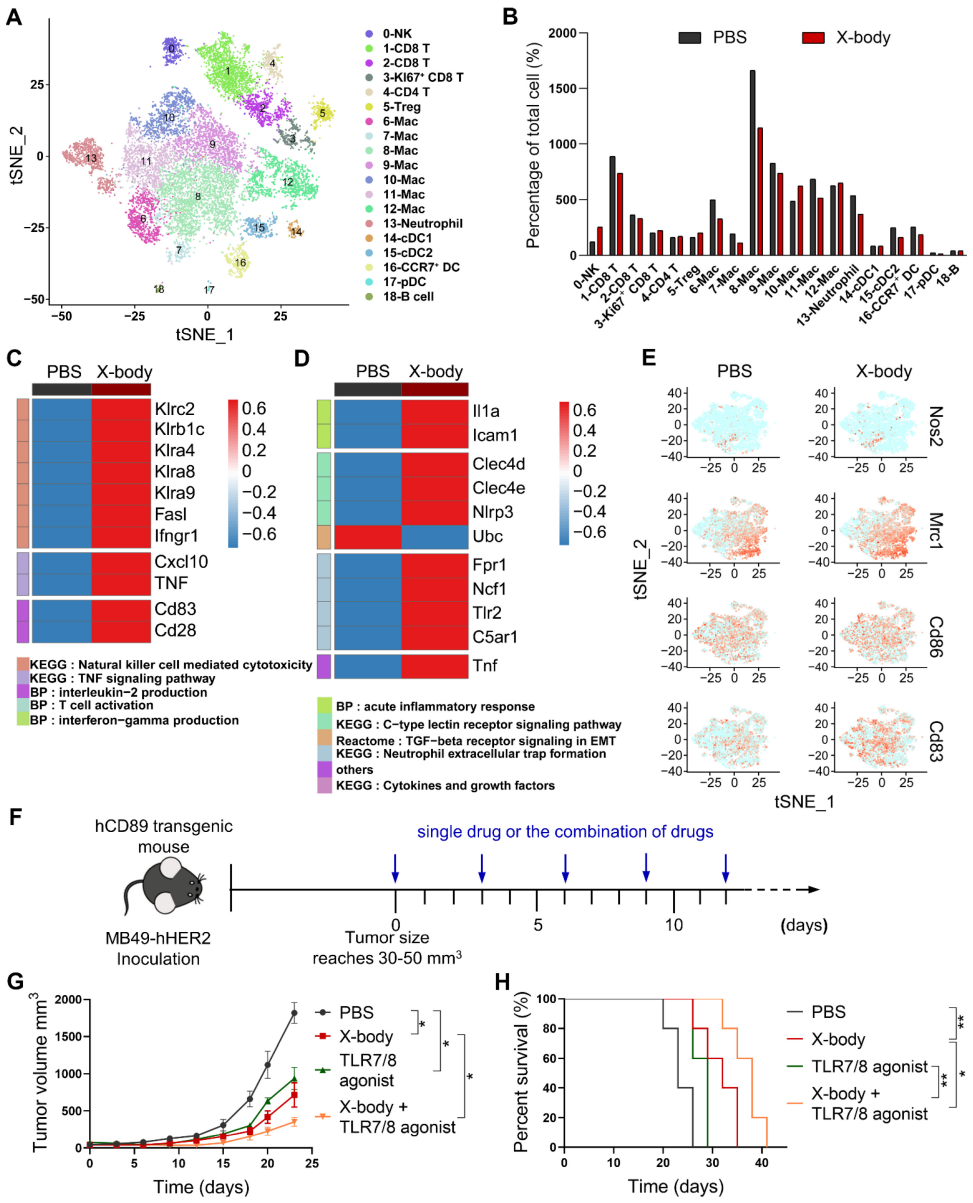
** Analysis of tumor-infiltrating immune cells by single-cell RNA sequencing. A)** Tumor infiltrating immune cells were isolated and subjected to single-cell RNA sequencing. The t-SNE plot showed the immune cells by scRNA-seq. **B)** Proportion of immune cells of different clusters in the total immune cells. **C-D)** Heatmap showing expression of genes in the selected pathways for NK cells (C) and neutrophils (D). **E)** t-SNE plot showing expression levels of selected genes defining M1/M2 phenotype of tumor-associated macrophages. **F)** Schematic of the treatment regimen of single drugs or the combination of drugs. MB49-HER2 cancer cells were subcutaneously implanted in human CD89 transgenic mice and the mice were treated by intraperitoneal injection of single drug such as PBS, trastuzumab X-body,TLR7/8 agonist or the combination of these drugs for 5 times. **G)** Tumor growth of mice treated with PBS, trastuzumab X-body,TLR7/8 agonist or the combination of the drugs. **H)** Kaplan-Meier survival curves of tumor-bearing mice. Data are presented as mean ± SEM. ** p ≤ 0.01, * p ≤ 0.05.

The tumor infiltrating immune cells were further analyzed using single-cell RNA-seq technology after 3 days after X-body treatment. Using cell lineage-specific markers, NK cells, neutrophils, and macrophages were identified from the tumor-infiltrating immune cells (Figure 6A, S7). X-body treatment increased the proportion of NK cells, but did not significantly change the proportions of neutrophils and macrophages in CD45^+^ cells (Figure 6B). X-body treatment upregulated the cytokines IFN-γ, *smad7*, *Icos*, *Stat3*, *TNF*, and *CXCL10* in NK cells, which are related to the activation of the TNF signaling pathway 18. In addition, NK activation receptors *Klra4 (Ly49d), Klra8 (Ly49h), Klra9, NKG2C (Klrc2), Klrb1c* 19, 20, and costimulatory receptors CD28 and LFA-1 (*Itgb2*) were increased and the inhibitory receptors *Klrg1, Klra1, Klrb1b, Klrd1*, and NKG2A (*Klrc1*) were downregulated upon X-body treatment (Figure 6C, S8). These results indicated that NK cells were activated by X-body treatment.

Activation of neutrophils by X-body treatment was observed as IL1α 21, Clec4d, and Clec4e 22, and TNF-α was upregulated compared to the vehicle control group (Figure 6D). Intercellular adhesion molecule 1(ICAM-1) which was associated with enhanced phagocytosis and reactive oxygen species generation of neutrophils was significantly upregulated. The immunosuppressive TGF-β receptor signaling pathway was downregulated. Nevertheless, X-body treatment promoted the NETosis pathway (such as Fpr1, Ncf1, Tlr2, and C5aR1), which is considered to exert a pro-tumoral effect. Therapeutic inhibition of NETosis could potentially blunt the unfavorable aspects of X-body-induced neutrophil activation, representing a potential axis to be exploited in combination with the X-body.

For deciphering the precise influence of tumor-associated macrophages (TAM) after X-body treatment, cells with high transcript of CD11b (*ITGAM*), F4/80 (*ADGRE1*) and CD115 (*Csf1r*) were defined as macrophages which accounted for nearly half of the tumor-infiltrating immune cells. Eight discrete clusters were identified with unique gene expression pattern (Figure S9A, B) and the top ten genes expression heatmap of these subpopulations was shown in Figure S10. Mac0 displayed a high level of major histocompatibility complex II (H2-Ab1, H2-Eb1), CD74 and interferon-induced transmembrane protein 1 (*Ifitm1*), suggesting that Mac0 was pro-inflammatory macrophages. The Mac1 was characterized with expression of complement B chain (C1qa and C1qb) and *Apoe*, suggesting that it is immunosuppressive population, Mac1 also expressed CCL12 and chemokine CXCL9 to play antitumor immune responses. Mac2 was typical M2-like macrophages featured with M2 macrophages biomarkers such as *Mrc1*, *Cbr2*, *F13a1*, *Maf* and* Timp2* 23. Mac3 was proliferating macrophages with expression of cell cycle factor (*Stmn1*), high transcript of Mki67, replication-dependent histone H1 family (*Hist1h1b*, *Hist1h2ae* and *Hist1h4d*) and mini-chromosome maintenance complex components (*Mcm3*, *Mcm5* and *Mcm6*). Mac4 expressed high level of Arg1, Mmp8, Cxcl3, Spp1 which together define immunosuppressive subsets of TAM 24. Mac5 was characterized by the expression of Chil3, placenta associated 8 (Plac8) and Ly6c2, which was recently reported in KPC pancreatic cancer mouse model 25. Mac6 expressed high level of interferon-induced genes (*Ifit2*, *Irf7* and *ifit1*) and chemokine CXCL10, indicating that Mac6 was proinflammatory macrophages. Mac7 cells were defined as doublets of macrophage and T cell.

We assessed whether any of the clusters correspond to canonical M1 or M2 classification (Nos2, CD83, CD86 for M1 and Mrc1 for M2) (Figure 6E). The macrophages were predominantly in an immunosuppressive M2-like state after X-body treatment. Therefore, reprogramming TAM from M2-like to M1-like presents the opportunity to sensitize the model to X-body.

Toll-like receptors (TLRs) play fundamental roles in the repolarization of macrophages and neutrophils. According to scRNA-seq data (Figure S11), TLR7 and TLR8 were expressed by macrophages and neutrophils. We combined trastuzumab X-body with TLR7/8 agonist (Figure 6F), the combination therapy improved tumor inhibition activity and produced 6 days and 9 days tumor growth delay compared to X-body and R848 treatment alone (Figure 6G, H).

### X-body shows no observable toxicity and exhibited a long serum half-life

**Figure 7:**
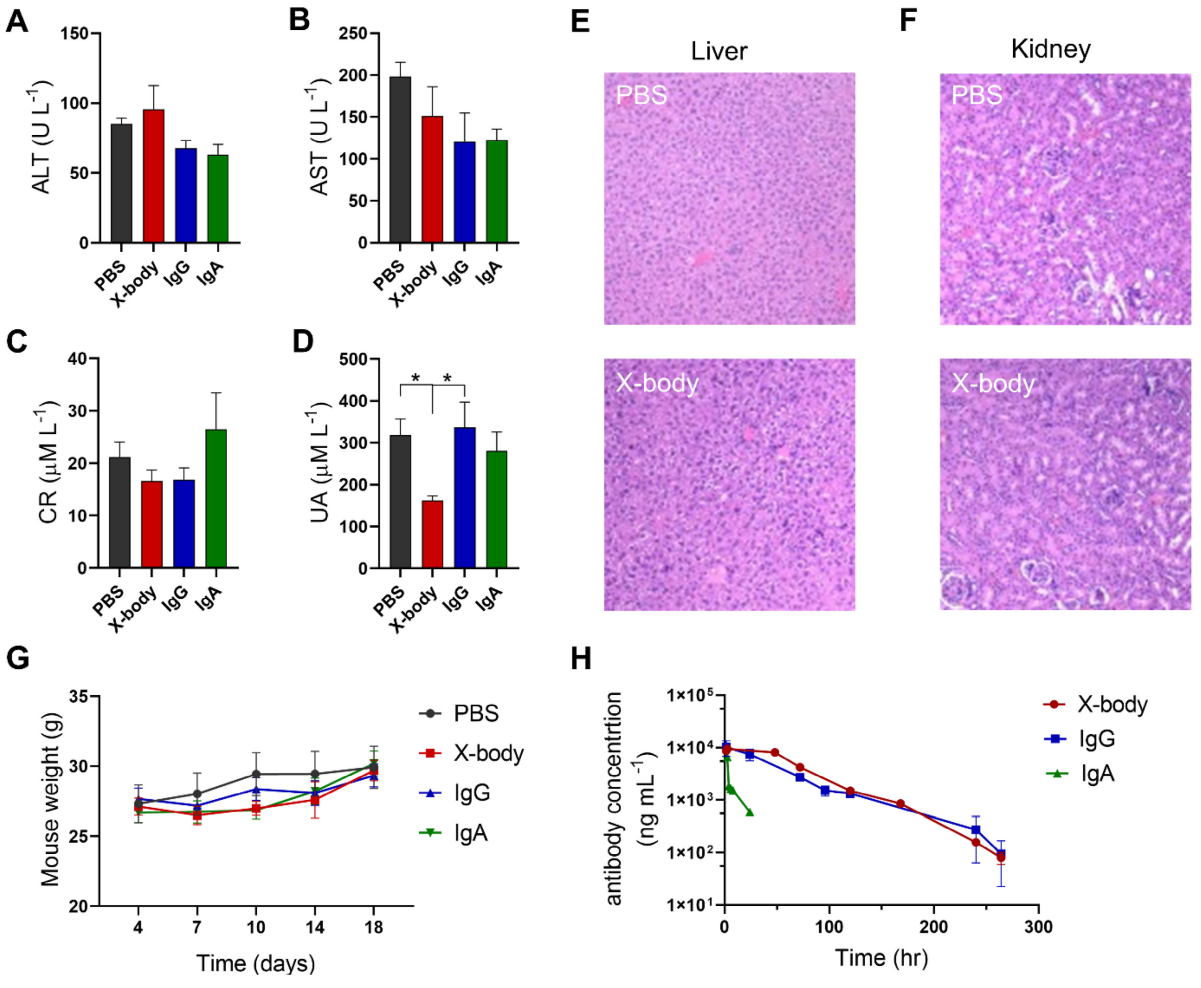
** Evaluation of toxicology and pharmacokinetics of X-body in mice. A-D)** Blood was collected three days after the last treatment and ALT (A), AST (B), CR (C) and UA (D) levels were analyzed. **E, F)** H&E staining of liver and kidney from MB49-HER2 tumor-bearing mice three days after the last treatment. **G)** Changes in body weight of MB49-HER2 tumor-bearing mice under different treatments. **H)** Pharmacokinetic profile. The hFcRn transgenic mice were injected with a single dose of antibody. Blood samples were collected at the indicated times. Serum levels for antibodies in mice were quantified by ELISA. The pharmacokinetic parameters were calculated by WinNonlin software.

Compared with PBS group, injection of X-body, IgA, or IgG did not change the levels of alanine aminotransferase (ALT), aspartate aminotransferase (AST), creatinine (Cr) (Figure 7A-C). Histological examination of the liver and kidney showed no histological alterations following X-body treatment (Figure 7E-F). X-body treatment did not cause weight changes compared to IgG, IgA, or PBS treatment (Figure 7G). The weights of the liver, spleen, and kidneys did not vary significantly between groups (Figure S12).

The *in vivo* half-life of the X-body was analyzed in human FcRn transgenic mice. As shown in Figure 7H, the half-life of X-body is similar to that of IgG and much longer than the half-life of IgA. All pharmacokinetic parameters of X-body are comparable to IgG (Table 2).

## Discussion

In this study, we described two unconventional antibody forms, X-body and square-body, both of which were designed to contain one IgG-Fc, one IgA-Fc, and two Fab fragments. The X-body maintains the function of each domain. X-body can engage with neutrophils, NK cells, and macrophages, thereby exhibiting superior anti-cancer potency both *in vitro* and* in vivo*. In terms of pharmacokinetics, the X-body can bind to FcRn through its IgG Fc, and has a serum half-life equivalent to that of IgG. In addition, injection of X-bodies into mice did not elicit observable liver and kidney toxicity. Although the square-body failed to show the desired activity, the concept of endowing antibodies with novel topology using the principles of molecular self-assembly could inspire the development of antibodies with novel features in the future.

Neutrophils have both IgA receptor CD89 and IgG receptors such as FcγRIIa and FcγRIIIb 26. However, IgA can induce neutrophils to kill tumor cells more effectively than IgG 27. This may be due to the higher affinity between IgA and FcαRI (Table 1 and Table S2). In addition, IgA-Fc binds to the extracellular domain of FcαRI in a 2:1 stoichiometry. It has been proposed that compared with the single immunoreceptor tyrosine-based activation motif (ITAM) of FcγRIIa, the higher stoichiometry of IgA to FcαR can activate up to four ITAMs, thereby triggering stronger Fc receptor signaling 28. Some studies provide evidence that human NK cells can also express FcαRI, which is involved in signal transduction and cell killing 29. In line with this, IgA could induce NK cell-mediated Ramos cell killing (Figure 2B). Therefore, IgA Fc of the X-body may further promote the activation of NK cells.

Prior to this study, several efforts were made to combine IgG Fc and IgA Fc with other groups, including IgGA 5, tandem IgG/IgA 4, 30, and TrisomAb 9. TrisomAb has achieved significant anti-tumor effects *in vitro* and *in vivo*. Similar to our X-body, TrisomAb eliminates cancer cells by recruiting NK cells, macrophages, and neutrophils as effector cells. TrisomAb adopts a bispecific antibody structure with one Fab replaced with an anti-FcαRI Fab. Unlike TrisomAb, our X-body features two Fab arms with bivalency for tumor antigens, which may lead to superior tumor cell targeting. However, whether the bigger size of X-bodies than trisomAb affected penetration into in solid tumors warrants further investigation in solid tumor models.

The effector function of the IgG isotype antibody can be modulated by engineering its IgG Fc affinity for FcγRs 31 and several antibodies with engineered IgG Fc have received approval. Many methods have been adopted to engineer IgG Fc, and the similar strategy can be applied to IgA engineering 32-35.

In summary, we have developed X-body platform that not only combines the full spectrum activity of IgG and IgA, but also displays good thermal stability and improved pharmacokinetics. The X-body can activate macrophages, neutrophils, and NK and is more potent to inhibit tumor growth than its IgG counterpart, which makes the X-body a potential option for cancer patients that do not benefit from current IgG-centered therapy.

## Supplementary Material

Supplementary figures and tables.

Supplementary Video S1-S3.

## Figures and Tables

**Table 1:** The equilibrium constants of Trastuzumab antibodies

	K_D_ (nM)				
	Her2	CD89	FcRn	FcγRIIIa^F158^	FcγRI
X-body	0.221	82.6	245	639	167
IgG	0.290	ND	995	320	113
IgA	0.204	58.8	ND	ND	272

ND: Not detectable.

**Table 2:** Pharmacokinetic parameters calculated by WinNonlin software

	Cmax (μg/mL)	AUC0-inf (h*μg/mL)	t1/2 (h)	Vz/F (mL)	CL/F (mL/h)
X-body	9.604 ± 0.167	777.23 ± 18.63	38.11 ± 1.50	10.56 ± 1.097	0.257 ± 0.006
IgG	10.01 ± 3.39	592.06 ± 74.28	41.81 ± 4.50	21.27 ± 8.29	0.341 ± 0.041
IgA	5.648 ± 1.432	47.29 ± 4.544	11 ± 1.71	72.03 ± 14.24	4.26 ± 0.42

## Abbreviations

TME: tumor microenvironment
CDC: complement-dependent cytotoxicity
ADCC: antibody-dependent cellular cytotoxicity
ADCP: antibody-dependent cellular phagocytosis
FcRn: neonatal Fc receptor
LDH: lactate dehydrogenase
PBMCs: peripheral blood mononuclear cells
TIL: tumor-infiltrating lymphocyte
PCA: principal component analysis
t-SNE: t-distributed stochastic neighbor embedding
KEGG: Kyoto Encyclopedia of Genes and Genomes
GSEA: gene-set enrichment analysis
SEC: size exclusion chromatography
NS-TEM: negative staining transmission electron microscopy
SPR: surface plasmon resonance
ELISA: enzyme linked immunosorbent assay
PMN: polymorphonuclear cells
TAM: tumor-associated macrophages
ICAM-1: intercellular adhesion molecule 1
*Ifitm1*: interferon-induced transmembrane protein 1
*Mcm*: mini-chromosome maintenance complex components
Plac8: placenta associated 8
ALT: alanine aminotransferase
AST: aspartate aminotransferase
Cr: creatinine
UA: uric acid
ITAM: immunoreceptor tyrosine-based activation motif
TAN: tumor-associated neutrophils
GM-CSF: granulocyte-macrophage colony-stimulating factor
TLRs: toll-like receptors
