# Supplementary material for: Self-assembly of X-shaped antibody to combine the activity of IgG and IgA for enhanced tumor killing
Source: Theranostics. 2022 Nov 14;12(18):7729–44. doi: 10.7150/thno.74903 (PMC9706586; doi:10.7150/thno.74903)
Supplement: Supplementary file 1 — Supplementary figures and tables. [file thnov12p7729s1.pdf]

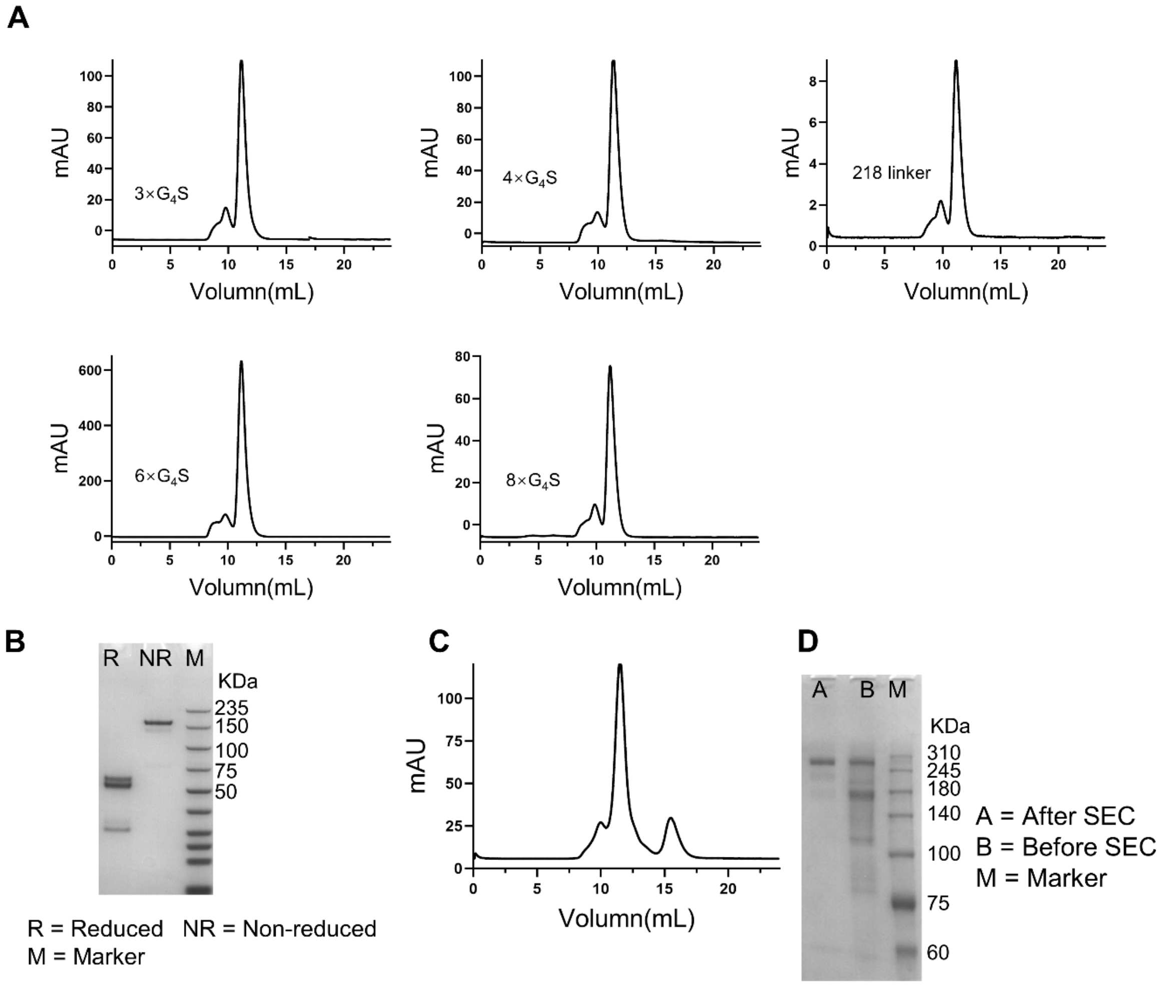

Figure S1. Purification of X-bodies and square-body.

A) Size-exclusion chromatography (SEC) results of X-bodies with different linkers (3×G_4S, 4×G_4S, 218 linker,6×G_4S, and 8×G_4S) between the light chain and the IgA Fc in the chimeric chain. B) Reducing and non-reducing SDS-PAGE results of anti-human CD20 X-body with 6×G_4S linker. C) SEC result of anti-human CD20 square-body with 6×G_4S linker. D) Non-reducing SDS-PAGE results of the square-body sample in (C).

A

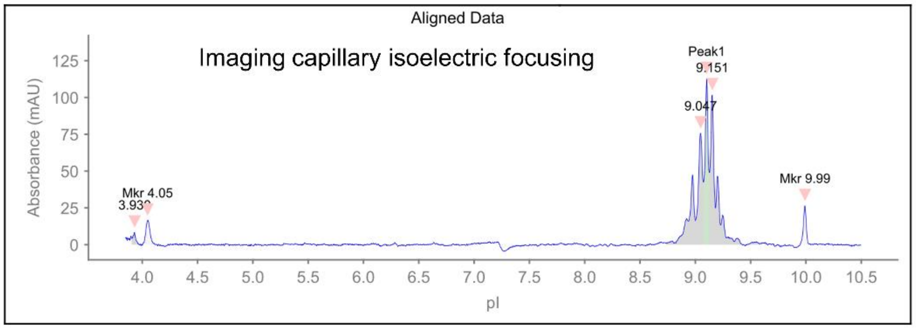

B

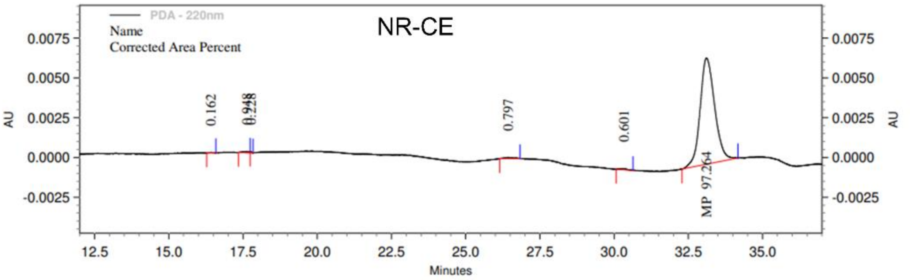

C

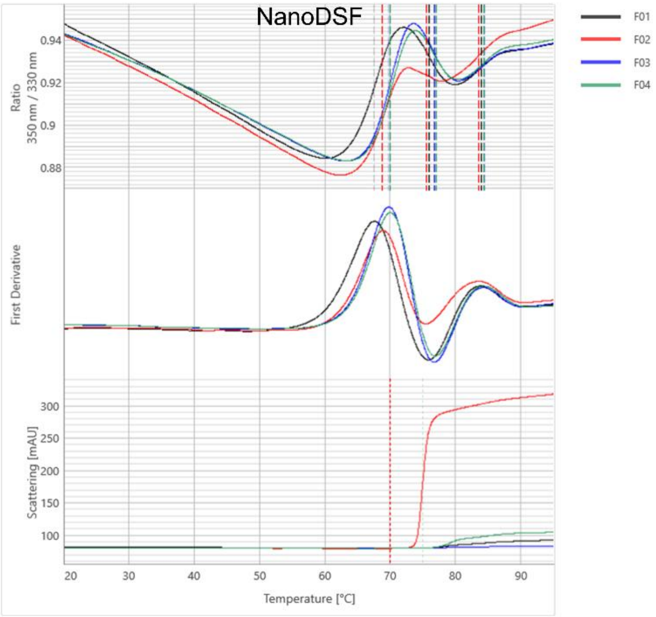

E

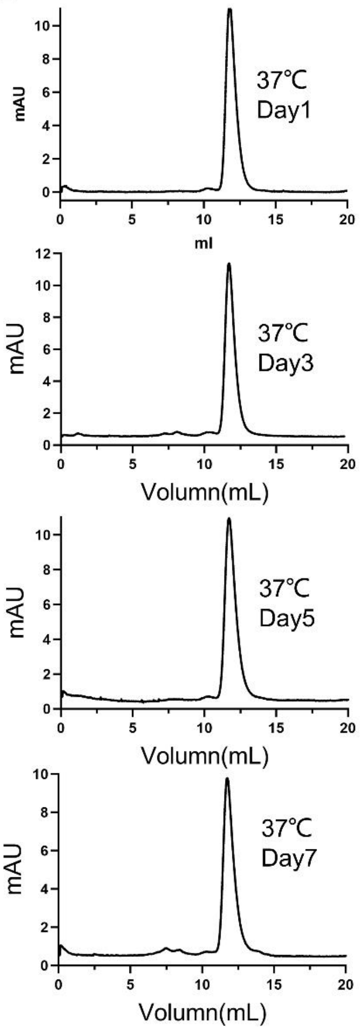

D

	T_m ℃	T_a ℃	Particle size(nm)	Concentration(mg mL^{-1})
**F01**	75.9	N/A	13.8	6.0
**F02**	75.6	70.0	15.3	4.6
**F03**	76.9	N/A	14.2	5.8
**F04**	77.0	75.0	14.3	14.4

Figure S2. characterization and stability assay of X-body.

A) The pI analysis of the anti-hCD20 X-body (pI = 9.103) by imaging capillary isoelectric focusing (iCIE). B) The purity analysis of the anti-hCD20 X-body (97.3%) by non-reducing capillary electrophoresis (NR-CE). The detection wavelength was 220 nm and the sample was injected for 20 s. C) The T_m of anti-hCD20 X-body in different buffers analyzed by the nano-format of Differential Scanning Fluorimetry (nanoDSF). F01 is PBS, F02 is HAC-NaAC buffer, F03 is CA-NaCA buffer and F04 is His-HCl buffer. D) The quantitative results of (C). E) The SEC profiles of X-body after 1, 3, 5 or 7 days of storage at 37°C.

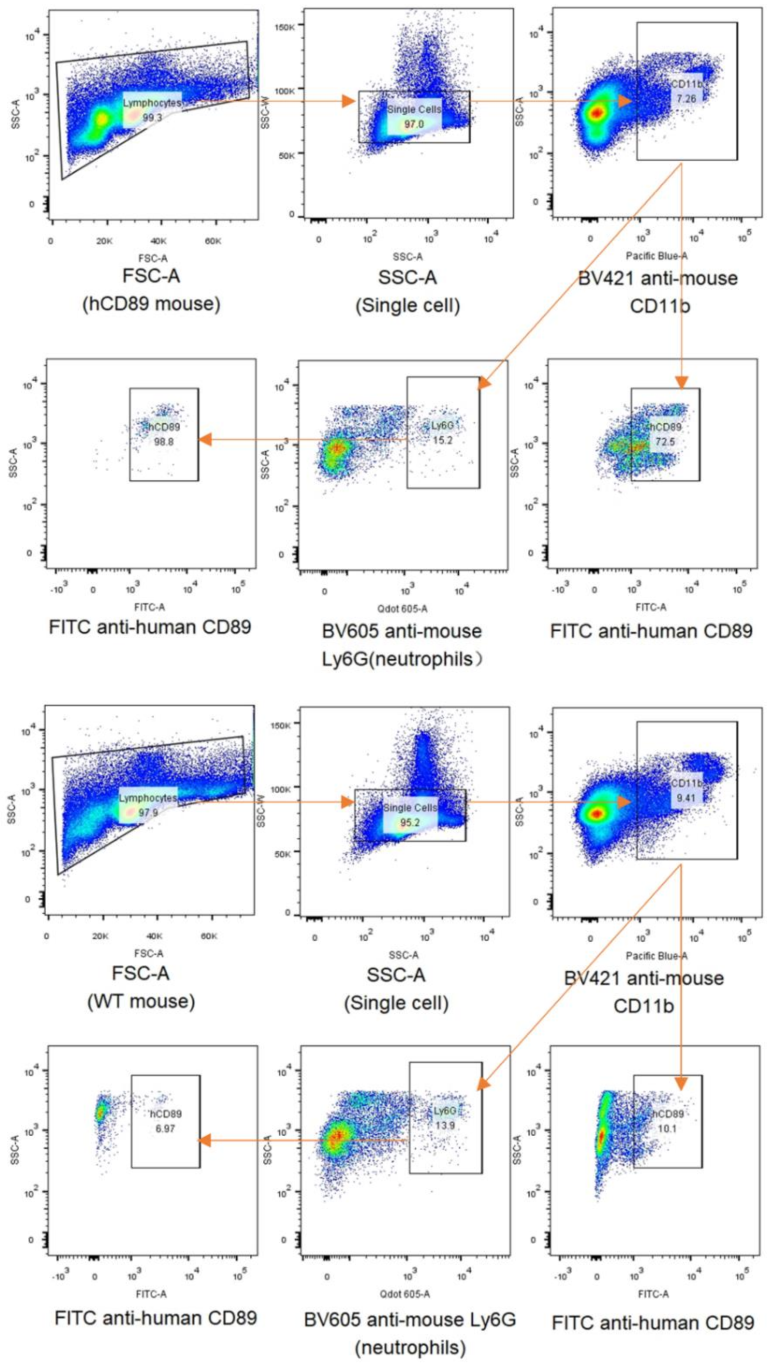

Figure S3. Validation of the human CD89 transgenic C57BL/6J mice by flow cytometry.

The figure depicts the gating strategies for identifying the expression of human CD89 in CD11b^{+} cells and neutrophils from the spleen of human CD89 transgenic C57BL/6J mice or the wild-type mice.

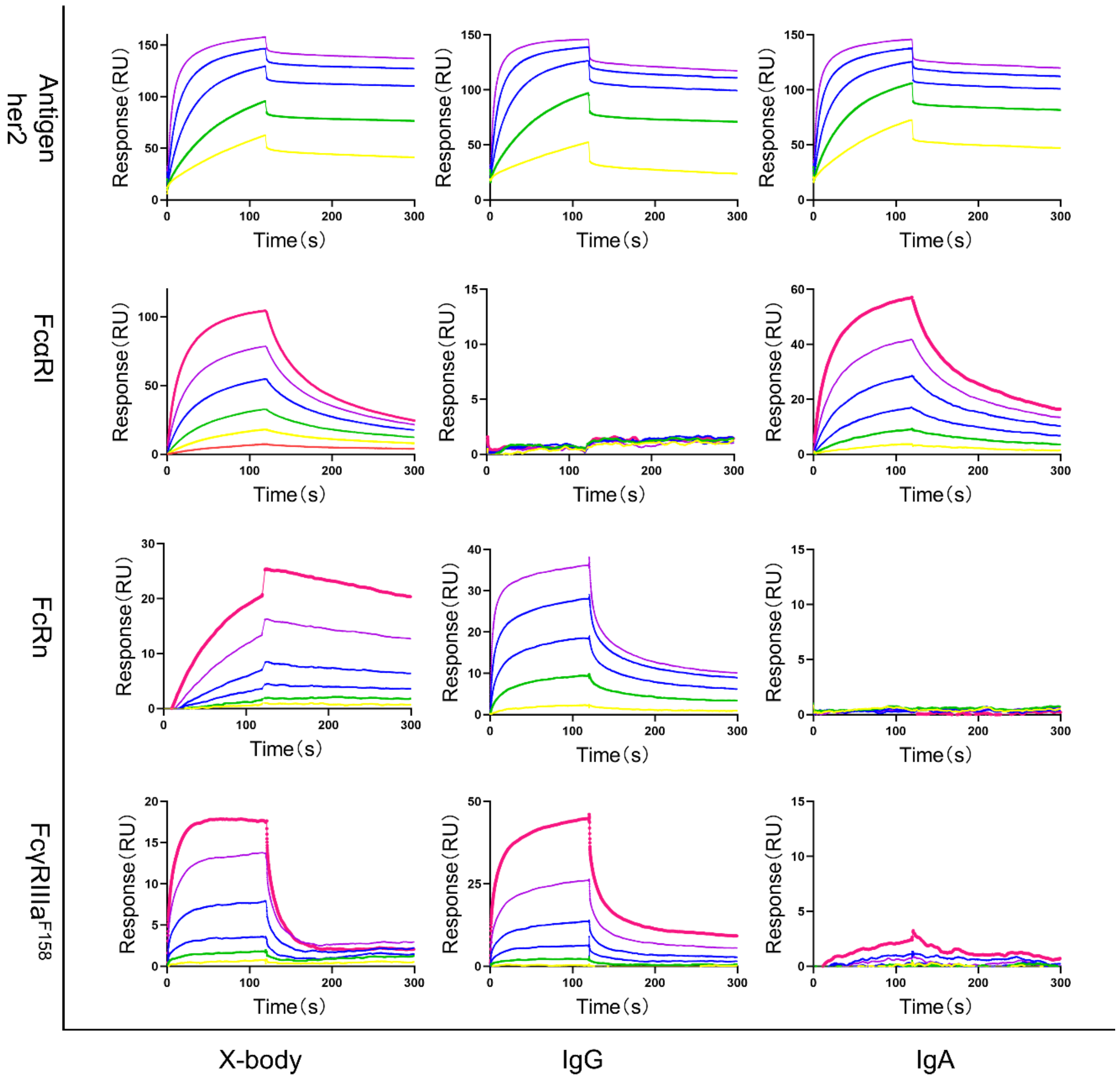

Figure S4. **Surface plasmon resonance analysis of trastuzumab antibodies binding to receptors and antigen (Her2).**

The binding of trastuzumab X-body to antigen HER2 and Fc receptors, such as CD89, FcRn and FcγRIIIa^{F158}, was evaluated by SPR. The IgA antibody used is monomeric IgA2 without the tailpiece for dimerization.

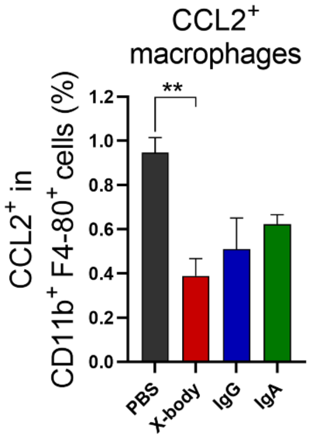

Figure S5. The expression of the chemokine CCL2 of tumor associated macrophages in MC38-HER2 tumor cell bearing syngeneic model by using flow cytometry.

MC38-HER2 cancer cells were subcutaneously implanted in human CD89 transgenic mice. When tumor reached 30-50 mm^3, the mice were treated with different version of trastuzumab. Tumor immune cells were isolated three days after 5 doses of drugs, stained with anti-CCL2 antibody and analyzed by flow cytometry. Data are presented as mean ± standard error of the mean (SEM). ** $p \leq 0.01$.

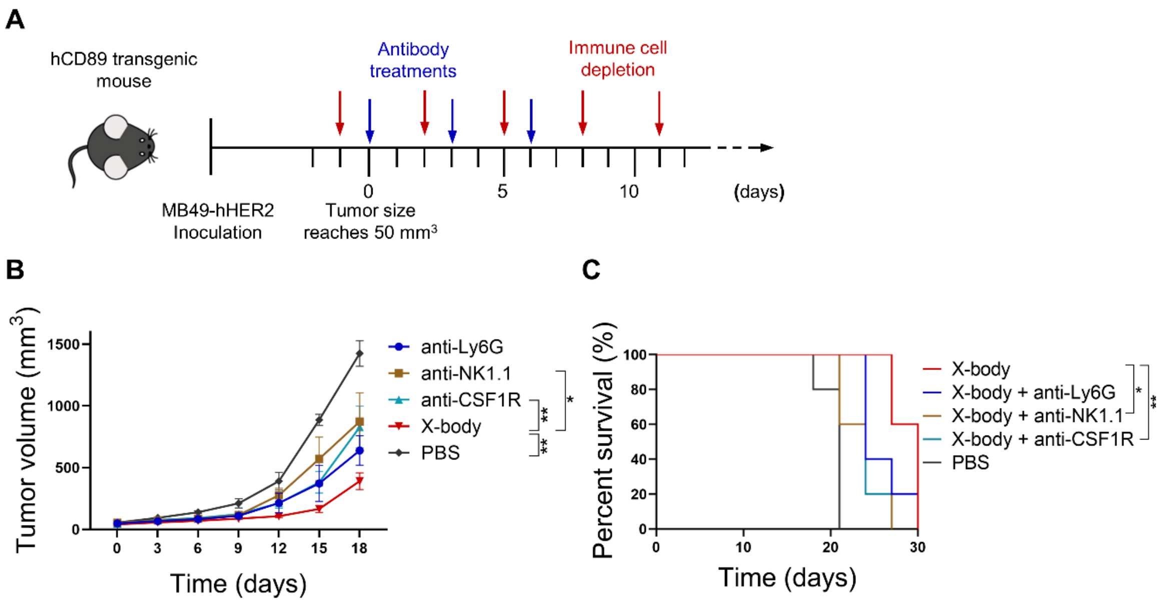

Figure S6. NK cells, macrophages, and neutrophils are required to eliminate tumors in Her2 expressing MB49 model.

A) Scheme of the treatment accompanied by immune cells depletion. MB49-HER2 cancer cells were subcutaneously implanted in human CD89 transgenic mice. Macrophages, neutrophils or NK cells were specifically depleted with anti-CSF1R, anti-Ly6G or anti-NK1.1 antibodies, respectively, prior to each dose of trastuzumab X-body. B) Tumor growth in the immune cell-depleted mice. C) Kaplan-Meier survival curves of the immune cell-depleted tumor-bearing mice. ** $p<0.01$, * $p<0.05$.

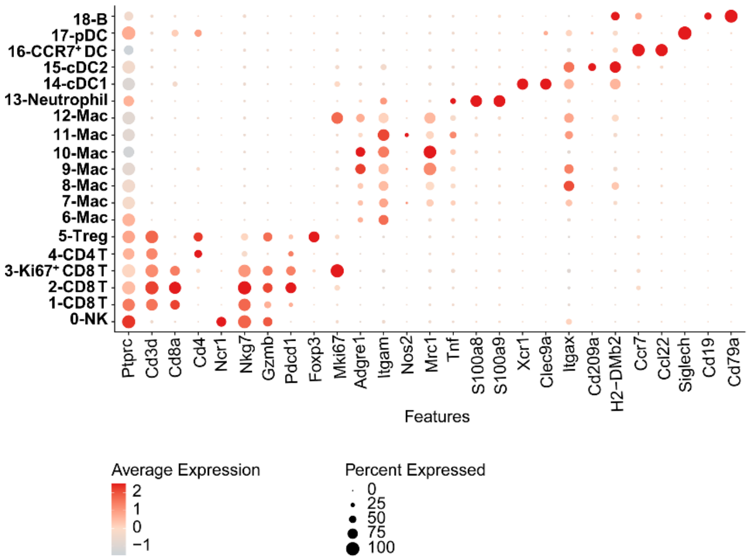

Figure S7. **Bubble heatmap showing marker genes across the 19 clusters from in Figure 6A.**

Dot size indicates the percent of expressing cells, and shades of color reflect normalized expression levels according to z-scores.

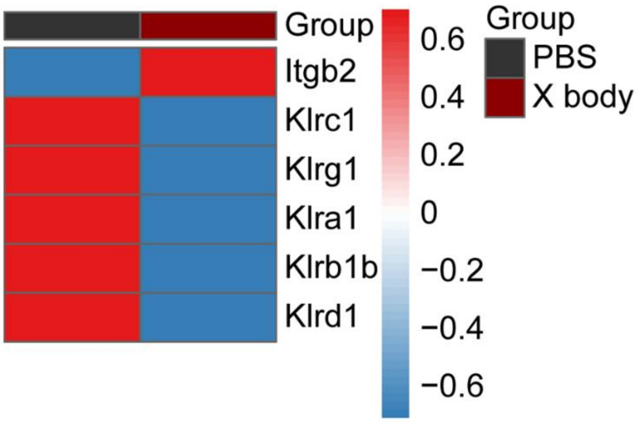

Figure S8. Heatmap showing expression patterns of selected pathway genes for NK cells by scRNA-seq.

Tumor infiltrating immune cells were isolated from mice in MB49-HER2 tumor models and subjected to single-cell RNA sequencing. Heatmap showed the inhibitory receptors of NK cells in PBS vehicle group (left) and X-body treatment group (right) by scRNA-seq.

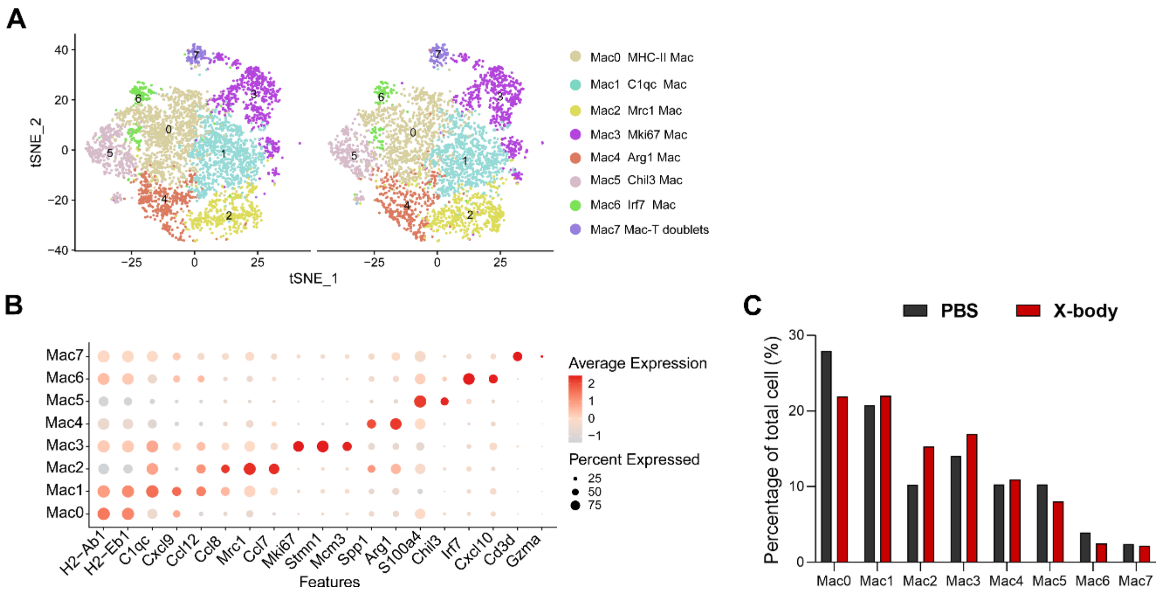

Figure S9. Characterization of tumor-associated macrophages by scRNA-seq.

A) t-SNE plot showing 8 subclusters of tumor-associated macrophages. B) Bubble heatmap showing marker genes across the 8 macrophage subclusters in (A). Dot size indicates the percent of expressing cells, and shades of color reflect normalized expression levels according to z-scores. C) Frequency of the 8 subclusters of tumor-associated macrophages.

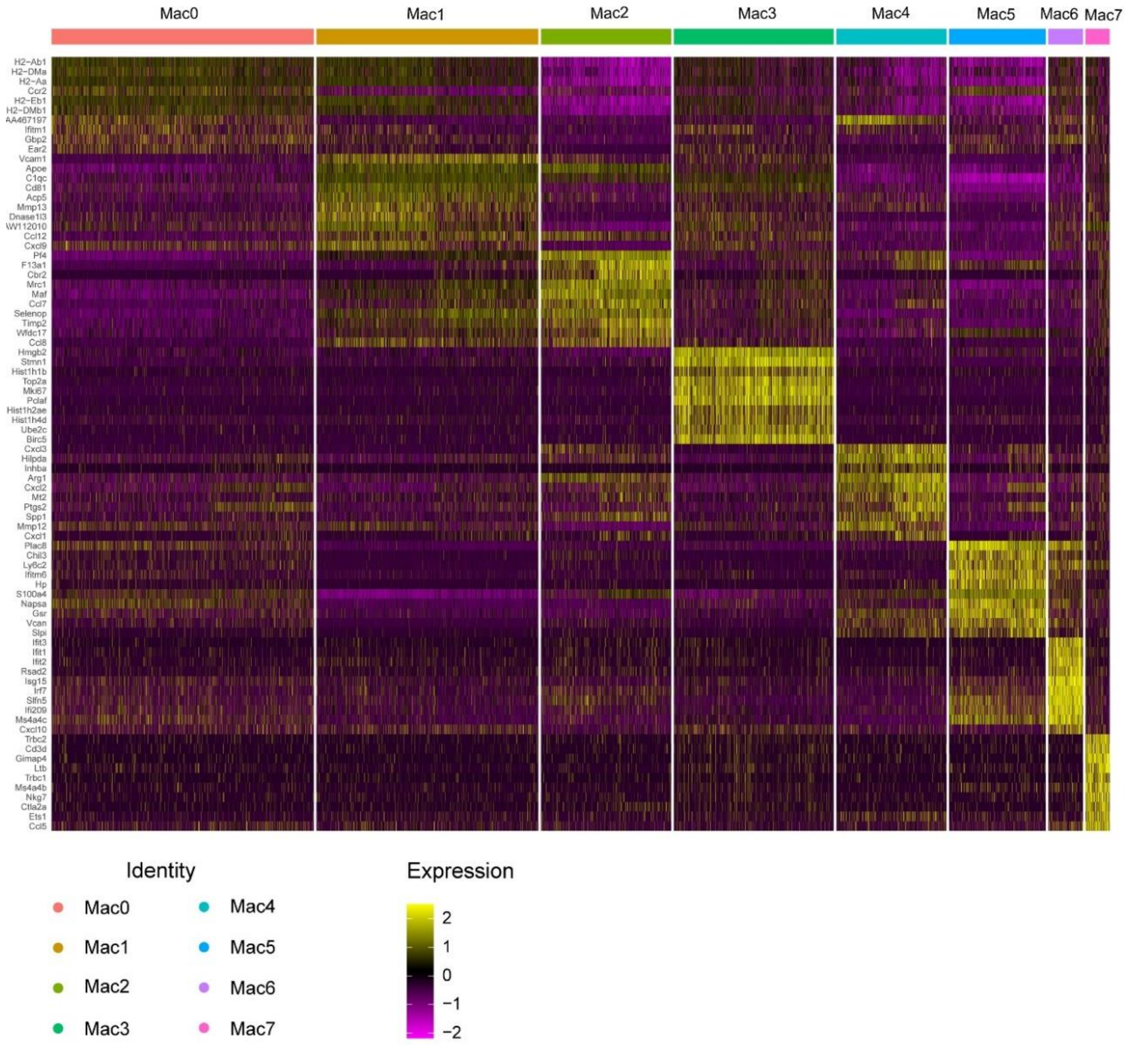

Figure S10. Top ten gene expression heatmap of tumor-associated macrophages from MB49-HER2 tumor models by scRNA-seq.

Tumor infiltrating immune cells were isolated from mice in MB49-HER2 tumor models and subjected to single-cell RNA sequencing. Heatmap showed the top ten gene expression levels of 8 subclusters of tumor-associated macrophages by scRNA-seq.

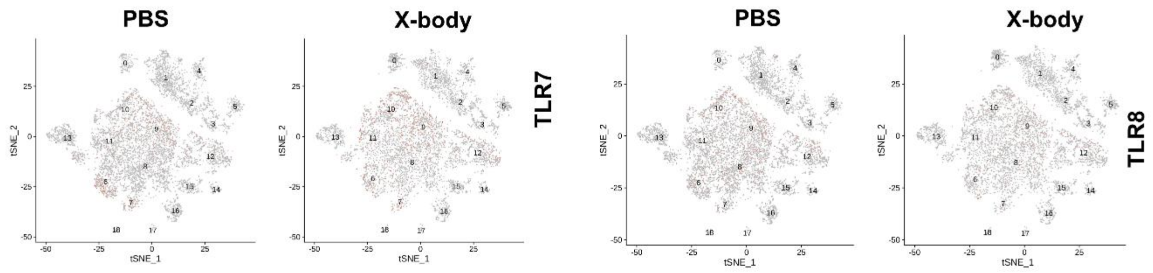

Figure S11. Expression levels of genes such as TLR7 and TLR8 from mice bearing MB49-HER2 tumors by scRNA-seq.

Tumor infiltrating immune cells were isolated from mice in MB49-HER2 tumor models and subjected to single-cell RNA sequencing. t-SNE plot showing expression levels of selected genes such as TLR7 and TLR8 of tumor-infiltrating immune cells in PBS vehicle group (left) and X-body treatment group (right) by scRNA-seq.

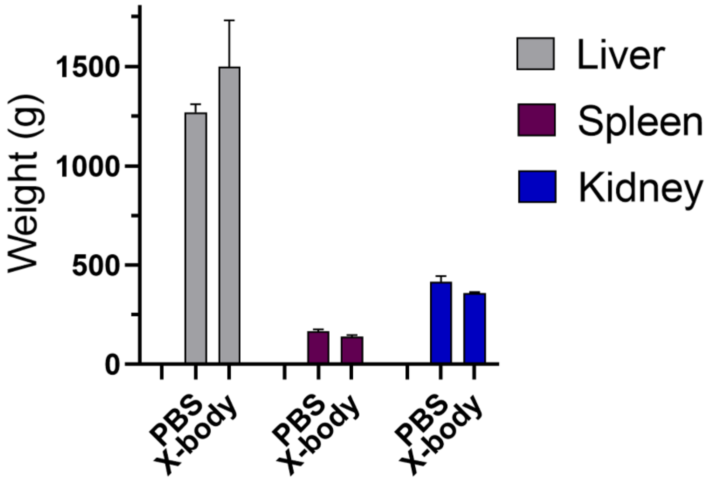

Figure S12. The weights of liver, spleen and kidney from the mice treated with PBS or trastuzumab X-body.

Human CD89 transgenic mice were treated with 10 mg/kg trastuzumab X-body or vehicle control. Liver, spleen and kidney were weighed.

Table S1. Amino acid sequences of Rituximab and Trastuzumab X-bodies.

**Rituximab X-body**
**Heavy chain:**<br>QVQLQQPGAELVKPGASVKMSCKASGYTFTSYNMHWVKQTPGRGLEWIGAIYPGNGDTSY<br>NQKFKGKATLTADKSSSTAYMQLSSLTSEDSAVYYCARSTYYGGDWYFNVWGAGTTVTVS<br>AASTKGPSVFPLAPSSKSTSGGTAALGCLVKDYFPEPVTVSWNSGALTSGVHTFPAVLQSSGL<br>YSLSSVVTVPSSSLGTQTYICNVNHKPSNTKVDKKVEPKSCDKTHTCPPCPAPELLGGPSVFLF<br>PPKPKDTLMISRTPEVTCVVVDVSHEDPEVKFNWYVDGVEVHNAKTKPREEQYNSTYRVVS<br>VLTVLHQDWLNGKEYKCKVSNKALPAPIEKTISKAKGQPREPQVYTLPPSRDELTKNQVSLT<br>CLVKGFYPSDIAVEWESNGQPENNYKTTPPVLDSDGSFFLYSKLTVDKSRWQQGNVFSCSVM<br>HEALHNHYTQKSLSLSPGK<br>**Chimeric chain:**<br>QIVLSQSPAILSASPGEKVTMTCRASSSVSYIHWFQQKPGSSPKPWIYATSNLASGVPVRFSGS<br>GSGTSYSLTISRVEAEDAATYYCQQWTSNPPTFGGGTKLEIKRTVAAPSVFIFPPSDEQLKSGT<br>ASVVCLLNNFYPREAKVQWKVDNALQSGNSQESVTEQDSKDSTYSLSSTLTLSKADYEKHK<br>VYACEVTHQGLSSPVTKSFNRGECGGGGSGGGGSGGGGSGGGGSGGGGSGGGGSVPPPPPC<br>CHPRLSLHRPALEDLLLGSEANLTCTLTGLRDASGATFTWTPSSGKSAVQGPPERDLCGCYSV<br>SSVLPGCAQPWNHGETFTCTAAHPELKTPLTANITKSGNTFRPEVHLLPPPSEELALNELVTLT<br>CLARGFSPKDVLVRWLQGSQELPREKYLTWASRQEPSQGTTTYAVTSILRVAAEDWKKGET<br>FSCMVGHEALPLAFTQKTIDRMAGK
**Trastuzumab X-body**
**Heavy chain:**<br>EVQLVESGGGLVQPGGSLRLSCAASGFNIKDTYIHWVRQAPGKGLEWVARIYPTNGYTRYA<br>DSVKGRFTISADTSKNTAYLQMNSLRAEDTAVYYCSRWGGDGFYAMDYWGQGTLVTVSSA<br>STKGPSVFPLAPSSKSTSGGTAALGCLVKDYFPEPVTVSWNSGALTSGVHTFPAVLQSSGLYS<br>LSSVVTVPSSSLGTQTYICNVNHKPSNTKVDKKVEPKSCDKTHTCPPCPAPELLGGPSVFLFPP<br>KPKDTLMISRTPEVTCVVVDVSHEDPEVKFNWYVDGVEVHNAKTKPREEQYNSTYRVVSVL<br>TVLHQDWLNGKEYKCKVSNKALPAPIEKTISKAKGQPREPQVYTLPPSRDELTKNQVSLTCL<br>VKGFYPSDIAVEWESNGQPENNYKTTPPVLDSDGSFFLYSKLTVDKSRWQQGNVFSCSVMH<br>EALHNHYTQKSLSLSPGK<br>**Chimeric chain:**

DIQMTQSPSSLSASVGDRVTITCRASQDVNTAVAWYQQKPGKAPKLLIYSASFLYSGVPSRFS
GSRSGTDFTLTISSLQPEDFATYYCQQHYTTPPTFGQGTKVEIKRTVAAPSVFIFPPSDEQLKSG
TASVVCLLNNFYPREAKVQWKVDNALQSGNSQESVTEQDSKDSTYSLSSTLTLSKADYEKH
KVYACEVTHQGLSSPVTKSFNRGECGGGGSGGGGSGGGGSGGGGSGGGGSGGGGSVPPPPP
CCHPRLSLHRPALEDLLLGSEANLTCTLTGLRDASGATFTWTPSSGKSAVQGPPERDLCGCYS
VSSVLPGCAQPWNHGETFTCTAAHPELKTPLTANITKSGNTFRPEVHLLPPPSEELALNELVTL
TCLARGFSPKDVLVRWLQGSQELPREKYLTWASRQEPSQGTTTYAVTSILRVAAEDWKKGE
TFSCMVGHEALPLAFTQKTIDRMAGK

Table S2. The equilibrium constants of Rituximab antibodies.

	K_D (nM)							
	**CD20**	**FcαRI**	**FcRn**	**FcγRI**	**FcγRIIa^{H131}**	**FcγRIIIA^{F158}**	**FcγRIIIA^{V158}**	**FcγRIIb**
**X-body**	8.04	0.291	0.0613	0.0102	0.0364	0.0216	0.0240	0.00422
**IgG**	3.74	ND	0.0391	0.0115	0.0548	0.0663	0.0603	0.00277
**IgA**	6.71	0.314	ND	0.00324	ND	ND	ND	ND

ND: Not detectable
